# Insights on mining the pangenome of *Sphingobacterium thalpophilum* NMS02 S296 from the resistant banana cultivar *Pisang lilin* confirms the antifungal action against *Fusarium oxysporum* f. sp. *cubense*

**DOI:** 10.3389/fmicb.2024.1443195

**Published:** 2024-09-19

**Authors:** B. R. Ajesh, R. Sariga, S. Nakkeeran, P. Renukadevi, N. Saranya, Saad Alkahtani

**Affiliations:** ^1^Department of Plant Pathology, Centre for Plant Protection Studies, Tamil Nadu Agricultural University, Coimbatore, Tamil Nadu, India; ^2^Department of Plant Biotechnology, Centre for Plant Molecular Biology & Biotechnology, Tamil Nadu Agricultural University, Coimbatore, Tamil Nadu, India; ^3^Department of Zoology, College of Science, King Saud University, Riyadh, Saudi Arabia

**Keywords:** Fusarium wilt, *Sphingobacterium thalpophilum* NMS02 S296, whole genome sequence, biocontrol agent, secondary metabolites, antifungal mechanisms

## Abstract

**Introduction:**

*Fusarium* wilt, caused by *Fusarium oxysporum* f. sp. *cubense* (*Foc*), poses a significant global threat to banana cultivation. Conventional methods of disease management are increasingly challenged, thus making it necessary to explore alternative strategies. Bacterial endophytes, particularly from resistant genotypes, are gaining attention as potential biocontrol agents. *Sphingobacterium thalpophilum*, isolated from the resistant banana cultivar *Pisang lilin* (JALHSB010000001-JALHSB010000029), presents an intriguing prospect for combating *Fusarium* wilt. However, its underlying biocontrol mechanisms remain poorly understood. This study aimed to elucidate the antifungal efficacy of *S. thalpophilum* NMS02 S296 against *Foc* and explore its biocontrol mechanisms at the genomic level.

**Methods:**

Whole genome sequencing of *S. thalpophilum* NMS02 S296 was conducted using next-generation sequencing technologies and bioinformatics analyses were performed to identify genes associated with antifungal properties. *In vitro* assays were used to assess the inhibitory effects of the bacterial isolate on the mycelial growth of *Foc*. To explore the biomolecules responsible for the observed antagonistic activity, metabolites diffused into the agar at the zone of inhibition between *Foc* S16 and *S. thalpophilum* NMS02 S296 were extracted and identified.

**Results:**

Whole genome sequencing revealed an array of genes encoding antifungal enzymes and secondary metabolites in *S. thalpophilum* NMS02 S296. In vitro experiments demonstrated significant inhibition of *Foc* mycelial growth by the bacterial endophyte. Comparative genomic analysis highlighted unique genomic features in *S. thalpophilum* linked to its biocontrol potential, setting it apart from other bacterial species.

**Discussion:**

The study underscores the remarkable antifungal efficacy of *S. thalpophilum* NMS02 S296 against *Fusarium* wilt. The genetic basis for its biocontrol potential was elucidated through whole genome sequencing, shedding light on the mechanisms behind its antifungal activity. This study advanced our understanding of bacterial endophytes as biocontrol agents and offers a promising avenue for plant growth promotion towards sustainable strategies to mitigate *Fusarium* wilt in banana cultivation.

## Graphical abstract

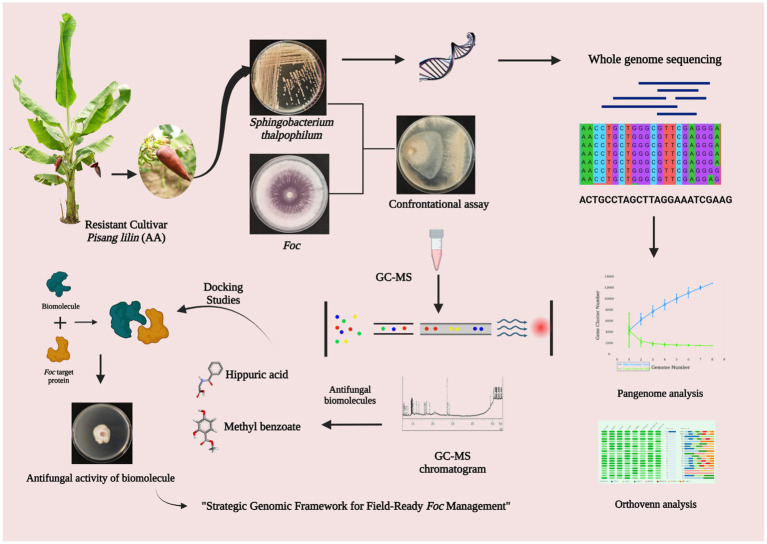

## 1 Introduction

In agricultural science, combating plant diseases and ensuring global food security is a persistent challenge. Fusarium wilt, caused by *Fusarium oxysporum* f. sp. *cubense* (*Foc*), remains one of the most devastating and economically significant plant diseases affecting banana (*Musa* spp.) and plantain (*Musa paradisiaca*) crops worldwide. This destructive pathogen infects the vascular system, leading to yellowing of fronds, drooping and wilting, reduced yields, and, in some cases, complete crop loss. Despite extensive research efforts to control this pathogen, finding sustainable and environmentally friendly solutions remains a top priority. The emergence of antimicrobial resistance and increasing concerns over the environmental impact of chemical pesticides have prompted scientists to explore novel approaches for managing Fusarium wilt. Endophytic bacteria, which reside within the plant tissues without causing harm, have been recognized as potential sources of novel bioactive compounds with antifungal properties (Hazra et al., 2024; Zotchev, 2024). These bacterial endophytes provide numerous beneficial effects, which include the production of metabolites, stimulation of plant growth through the synthesis of phytohormones, induction of the plant’s innate immune response, and protection against various plant pathogens (Nakkeeran et al., 2021; Santoyo et al., 2016).

In this context, the spotlight is on the microorganism *Sphingobacterium thalpophilum* NMS02 S296, a versatile endophytic bacterium isolated from the bracts of resistant banana cultivar., *Pisang Lilin* (AA). *Sphingobacterium* are rod-shaped Gram-negative genus that exhibits remarkable metabolic versatility, relying on aerobic respiration and lacking motility (Xu et al., 2017; Sun et al., 2015). It has the potential to survive in various extreme environments, and they can be found in a wide range of habitats, including freshwater, compost, soil, activated sludge, and plants. Some *Sphingobacterium* strains have been witnessed for the promotion of plant growth (Ahmed et al., 2014; Mehnaz et al., 2007). However, limited research has been devoted to investigating the potential of *Sphingobacterium* strains in suppressing fungal pathogens. One school of thought has confirmed the presence of an extracellular chitosanase produced by *Sphingobacterium multivorum* (Matsuda et al., 2001). Genome analysis of *Sphingobacterium psychroaquaticum* strain SJ-25 confirmed the presence of genes involved in the production of 2, 3-butanediol, and salicylic acid, which can induce systemic resistance in the host plant (Xu et al., 2020). Our isolate, *S. thalpophilum* NMS02 S296 possessed remarkable antagonistic properties against *Foc*, offering hope for a natural and sustainable solution to the management of Fusarium wilt of banana. This study presents a comprehensive investigation into the complete genome sequence of *Sphingobacterium thalpophilum* NMS02 S296 and its potential as a biological agent against *Foc*. By unraveling the genomic secrets of this bacterium and exploring its interactions with the pathogen, we aim to shed light on its antifungal mechanisms and assess its potential as a biocontrol agent for managing *Fusarium* wilt.

The complete genome sequencing of *S. thalpophilum* NMS02 S296 promises to unveil an array of genes, pathways, and metabolites with the potential to suppress *Foc* and enhance plant health. Additionally, the study will delve into the production of secondary metabolites, which may play a vital role in antagonistic activity. Understanding the genomics of NMS02 S296 will allow us to decipher the molecular underpinnings of its interactions with *Foc*, shedding light on the mechanisms that enable this bacterium to thrive in the presence of the pathogen. Our research will encompass *in vitro* experiments to investigate the antifungal activity of *S. thalpophilum* NMS02 S296 against *Foc*. By utilizing state-of-the-art molecular biology techniques and bioinformatics tools, we aim to provide insights into the specific genes and metabolic pathways involved in the antagonistic effect of NMS02 S296.

As we navigate the dynamic landscape of agricultural science, this study stands at the intersection of genomics, biocontrol, and plant pathology, offering a comprehensive exploration of *S. thalpophilum* NMS02 S296 as a potential ally in the battle against *Fusarium* wilt of banana. We hope that the insights gleaned from this research will pave the way for innovative and sustainable solutions to mitigate the impact of this destructive pathogen, securing the future of banana and plantain cultivation and addressing the broader challenges of food security and agricultural sustainability.

## 2 Materials and methods

### 2.1 Source and molecular confirmation of microorganisms

The pathogen, *Foc* S16 (Accn. No: PP239076) was isolated from the *Fusarium* wilt-infected Rasthali (AAB) banana variety. The pathogen was confirmed as *Foc* as per the protocol described by Saravanan et al. (2022). Sixteen bacterial endophytes obtained from the Culture Collection Centre, Department of Plant Pathology, Tamil Nadu Agricultural University, Coimbatore, Tamil Nadu, India, were screened against *Foc* S16 (Supplementary Table S1).

### 2.2 Antagonistic activity of bacterial endophytes against *Foc*

The effectiveness of bacterial endophytes in inhibiting the growth of *Foc* S16 was evaluated through an *in vitro* confrontational assay. A mycelial disc with a diameter of 9 mm, obtained from a 7 day-old culture of *Foc* S16, was placed on one side of a sterile Petri plate containing PDA medium. At a distance of 1 cm away from the periphery, all the bacterial endophytes (16 Nos.) were streaked on the medium, positioned directly opposite to the mycelial disc to assess the antifungal efficacy. The plates were incubated at 28 ± 2°C for 7 days. Following the incubation, the zone of inhibition against *Foc* S16 was measured. The experiment was repeated three times, with each replication consisting of 10 Petri plates. The Petri plate exclusively with the pathogen alone was maintained as an untreated control. The percent inhibition of mycelial growth of bacterial antagonists over untreated control was calculated using the formula: Percent Growth inhibition = (C − T)/C × 100.

Where, C = Mycelial growth of the pathogen in (mm) untreated control plate.

T = Mycelial growth of the pathogen in (mm) dual plate.

### 2.3 Metabolite extraction and identification of biomolecules from the zone of inhibition produced by *S. thalpophilum* NMS02 S296 against *Foc* S16

The study involved co-culturing of antagonistic bacteria with the pathogen to examine the variably expressed VOCs/NVOCs during di-trophic interactions. Bioactive VOCs/NVOCs which are diffused into the agar at the zone of inhibition between *Foc* S16 and *S. thalpophilum* NMS02 S296 were extracted by following the procedure outlined by Cawoy et al. (2015). The extracted biomolecules from the diffused agar were then subjected to metabolic profiling using Gas Chromatography–Mass Spectrometry (GC–MS) to identify the specific compounds present. This allowed for the precise characterization of the bioactive compounds as per the methodology described by Saravanan et al. (2022).

### 2.4 Molecular docking and experimental validation of antifungal activity of biomolecule in wet lab conditions

In the exploration of biomolecules produced by strain NMS02 S296 during its interaction with *Foc* S16, an in-silico docking analysis was employed. Based on a literature survey and analysis, fourteen antifungal protein targets in *Foc* were identified for their critical roles in fungal metabolism, pathogenicity, and survival. These proteins were modeled using SWISS-MODEL, Phyre2, and ROBETTA software by Nayana et al. (2023) and utilized to assess the binding strength and conformation of biomolecules in comparison with the widely used fungicide tebuconazole, which served as the positive control in this study. The different target proteins of *Foc* used for docking as follows: G-protein-β-subunit 1 (FGB1) (Soundararajan et al., 2011), GTPase activating protein (RHO1) (Martínez-Rocha et al., 2008), 5′ → 3′ Exoribonuclease 2 (XRN2) (Maldonado Bonilla and Calderón-Oropeza, 2018), Fusarium transcription factor 1 (FTF1), Velvet (Ghag et al., 2014), Resistance to the lethality of MKK1P386 (RLM1) (Ding et al., 2020), C-4 sterol methyl oxidase (ERG25) (Deng et al., 2015), Six Gene Expression 1 (SGE1) (Gurdaswani et al., 2020), MAP kinase kinase 2 (MKK2) (Ding et al., 2015), Secreted in xylem 1 (SIX 1), Secreted in xylem 6 (SIX 6), Secreted in xylem 8 (SIX 8), Secreted in xylem 10 (SIX 10), and Secreted in xylem 13 (SIX 13) (Carvalhais et al., 2019) (Supplementary Figure S1). Docking experiments were conducted using the AutoDock Vina module in PyRx 0.8 software (Dallakyan and Olson, 2015). Binding site pockets for the targets were defined using the Computed Atlas Topography of Proteins (CASTp 3.0) server (Tian et al., 2018). Visualization of the docked structures was performed using BIOVIA Discovery Studio Client 2021. To generate a standardized comparison of binding affinities across different biomolecules and target proteins, a heatmap was generated using Clustalvis heatmap tool which utilizes z-score normalization to transform the binding energy to a −3 to 3 scale (Metsalu and Vilo, 2015).

The biomolecule with higher binding affinity was selected and purchased in its pure form from Sigma Aldrich and later screened for its antifungal effectiveness against *Foc* through the poison food technique. For this, different concentrations of the biomolecule, namely 100 ppm, 250 ppm, 500 ppm, 750 ppm, and 1,000 ppm, were prepared from a 10,000 ppm stock solution. Additionally, an untreated control was maintained separately. Petri plates were filled with 15 mL of media containing various concentrations of the compound and allowed to solidify. Subsequently, a 9 mm mycelial disc of a 7 days-old culture of *Foc* was placed at the center of each plate and incubated at 28 ± 2°C for 5 days. The colony growth was recorded after 7 days of incubation. The mycelial growth of *Foc* was measured across different treatments and compared with the untreated control to determine the percent reduction in mycelial growth to the untreated control.

### 2.5 Comprehensive genome analysis and genome assembly

The genomic DNA of *S. thalpophilum* NMS02 S296 was extracted using the Quick-DNA Fungal/Bacterial kit (D6005) and then underwent whole-genome sequencing using the illumina platform. To conduct a thorough analysis of the S296 isolate, the sequenced reads were submitted to PATRIC and processed using the TORMES-1.0 Unicyclker tools. Utilizing the TORMES-1.0 platform, the reads were further processed and analyzed.

### 2.6 Genome annotation and assembly

The genome of *S. thalpophilum* NMS02 S296 was annotated using the RAST tool kit (RASTtk) and genetic code 11. This annotation was performed by comparing with other genomes in the PATRIC database to identify specific genes of interest, categorize them into functional subsystems, and a phylogenetic tree was constructed for analysis. The proteins with functional assignments were annotated based on the protocol established by Schomburg et al. (2017). Gene Ontology (GO) assignments were carried out following the protocol described by Ashburner et al. (2000). Mapping to KEGG pathways was performed using the protocol described by Kanehisa (2017). Annotation of protein families in the NMS02 S296 genome was conducted according to the guidelines outlined by Davis et al. (2016). The draft genome was screened against virulence factors using Abricate and PATRIC to identify virulence genes, with hits having 90% coverage and identities below 90% excluded. Subsystem analysis was performed to gain insights into the involvement of specific sets of proteins that contribute to distinct biological processes or structural complexes. Additionally, the annotation included the identification of regions in the NMS02 S296 genome that code for secondary metabolites using the “relaxed” antiSMASH 6.0.1 strictness.

### 2.7 Comparative genome analysis

The different isolates related to the *S. thalpophilum* NMS02 S296 strain, including the assembled contigs of the test isolate NMS02 S296 and 7 selected *Sphingobacterium* spp. genomes underwent a comprehensive characterization using M1CR0B1AL1Z3R (Avram et al., 2019). This analysis involved gene annotation, ortholog detection, sequence alignment, and phylogeny reconstruction. To extract the putative open reading frames (ORFs) from each genome, Prodigal was used in its “normal” mode (Hyatt et al., 2010). This approach employed unsupervised machine learning to identify protein-coding ORFs. A homology search was then conducted, where each ORF was compared against all other ORFs in the database. To detect high-confidence orthologous groups, the Markov Cluster (MCL) algorithm was employed with default parameters, including an inflation parameter of 2.0 (Li et al., 2003).

### 2.8 Phylogenetic analysis

PATRIC incorporates reference and representative genomes in its comprehensive genome analysis report and it was used for phylogenetic analysis. The process of identifying the closest reference and representative genomes involves Mash/MinHash (Stamatakis, 2014). From these selected genomes, the system extracts PATRIC global protein families (PGFams) to determine the phylogenetic positioning of the genome in question (Davis et al., 2016). To achieve it, protein sequences from PGFams were aligned using MUSCLE, and the corresponding nucleotide sequences were mapped to the protein alignment (Edgar, 2004). These amino acid and nucleotide alignments are concatenated into a comprehensive data matrix. RaxML, with fast bootstrapping, was employed to analyze this matrix, and support values were generated for the resulting tree. The RAxML software was utilized with default parameters, including an LG replacement matrix, a discrete gamma distribution with four categories, and an invariant category (LG + G + I) to account for variation in rates among sites (Le et al., 2008).

### 2.9 Pangenome analysis of *Sphingobacterium thalpophilum*

The pangenome analysis of *Sphingobacterium thalpophilum* was carried out using available genomes of related strains obtained from the NCBI database. Genomic sequences of four *Sphingobacterium thalpophilum* strains, specifically *S. thalpophilum* NMS02 S296, *S. thalpophilum* NCTC11429, *S. thalpophilum* BAA-1094, and *S. thalpophilum* YX-3, were retrieved for comparative analysis. The pangenome analysis was performed utilizing the PGAP webserver to identify core genes (present in all strains), accessory genes (present in some strains), and unique genes (specific to *S. thalpophilum* NMS02 S296). The clustering of all nucleic acid and protein sequences was conducted via the GeneFamily (GF) method within the pan-genome analysis pipeline (PGAP) (Zhao et al., 2012) employing default criteria. Additionally, a phylogenetic tree was generated utilizing the pan-based neighbor-joining method inorder to provide a detailed representation of the genetic diversity and evolutionary history of these strains through a phylogenomic approach.

## 3 Results

### 3.1 Antagonistic efficacy of bacterial endophytes against *Foc*

**Figure 1:**
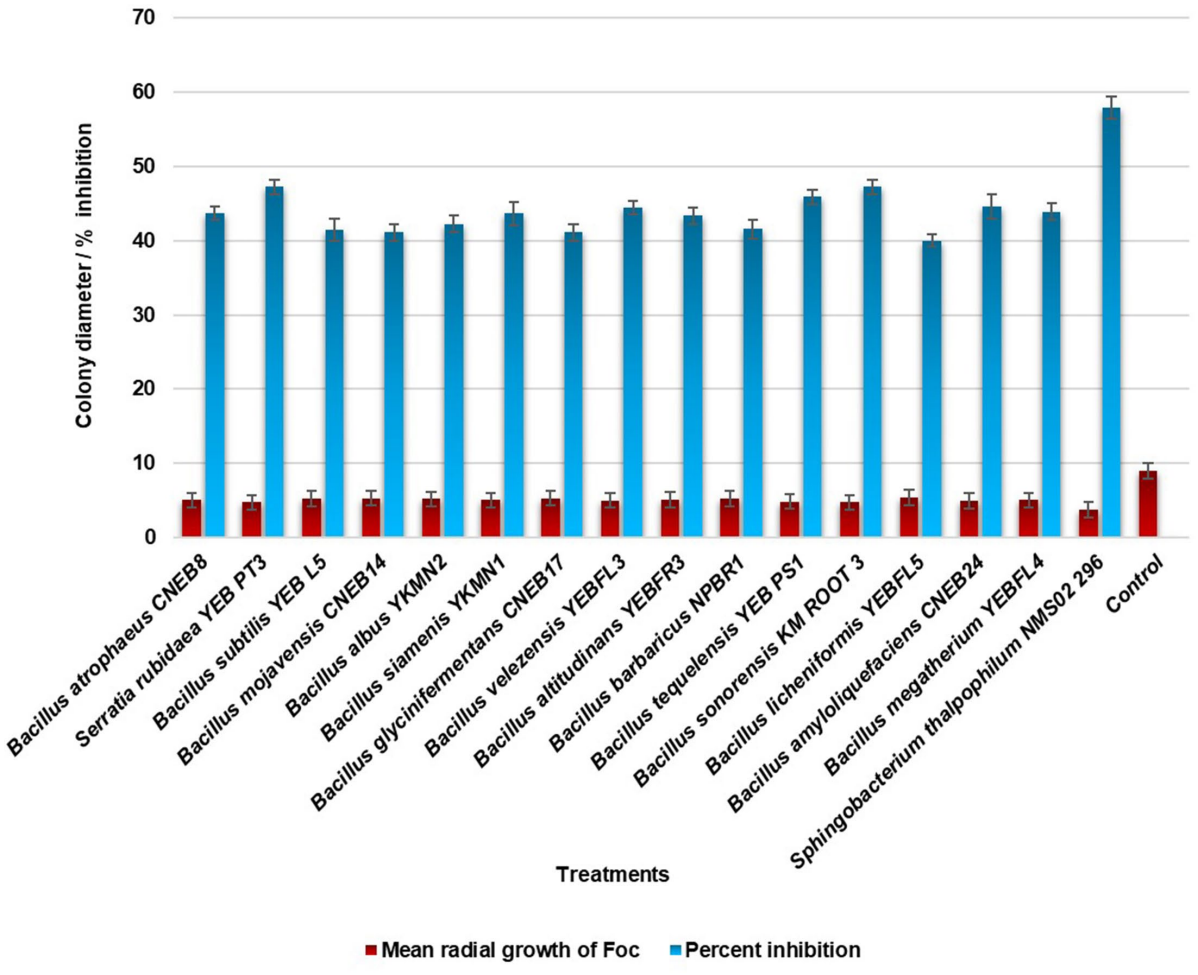
*In vitro* antagonistic activity of bacterial endophytes against *Foc*.

In the evaluation of the antagonistic potential of bacterial endophytes against *Foc*, 16 isolates were tested and compared. Notably, *S. thalpophilum* NMS02 S296, derived from the rhizosphere soil of the resistant banana genotype *Pisang lilin*, exhibited superior antifungal activity with an inhibition percentage of 57.88% against *Foc* when compared to other isolates (Figure 1; Supplementary Figure S2).

### 3.2 Identification of biomolecules from the zone of inhibition produced by *S. thalpophilum* NMS02 S296 against *Foc*

The GC–MS analysis of the biomolecules extracted from the zone of inhibition during the interaction of NMS02 S296 (bacterial antagonist) with *Foc* revealed the diversity of biomolecules in the zone of inhibition which was different from those produced by *Foc* alone or by NMS02 S296 alone (Supplementary Figures S3–S5). Compounds detected from the dual culture of *Foc* S16 and NMS02 S296 bacteria included 2-methyl caproate, 1,3-cyclopentanediol, dihydroxy dimethyl furanone, 3-methyl-2-oxovaleric acid, 5-aminovaleric acid, 4H-pyranone, dihydroxyacetone, norvaline, acetoacetic acid, aminobutyric acid, succinic acid, hippuric acid, methyl-2,4-dihydroxybenzoate, methyl linolelaidate, ureidopropionic acid, 4-hydroxybutyric acid, palmitoleic acid, linoleic acid, and methyl myristoleate. The compounds produced by NMS02 S296 alone were cresol, oleic acid, 2-aminooctanoic acid, tiglylglycine, octadecanoic acid, acetamide, methyl erucate, cinnamic acid, isoleucine, arachidonic acid, nonadecanamide, and squalene.

### 3.3 Molecular docking and experimental validation of antifungal activity of biomolecule in wet lab conditions

**Figure 2:**
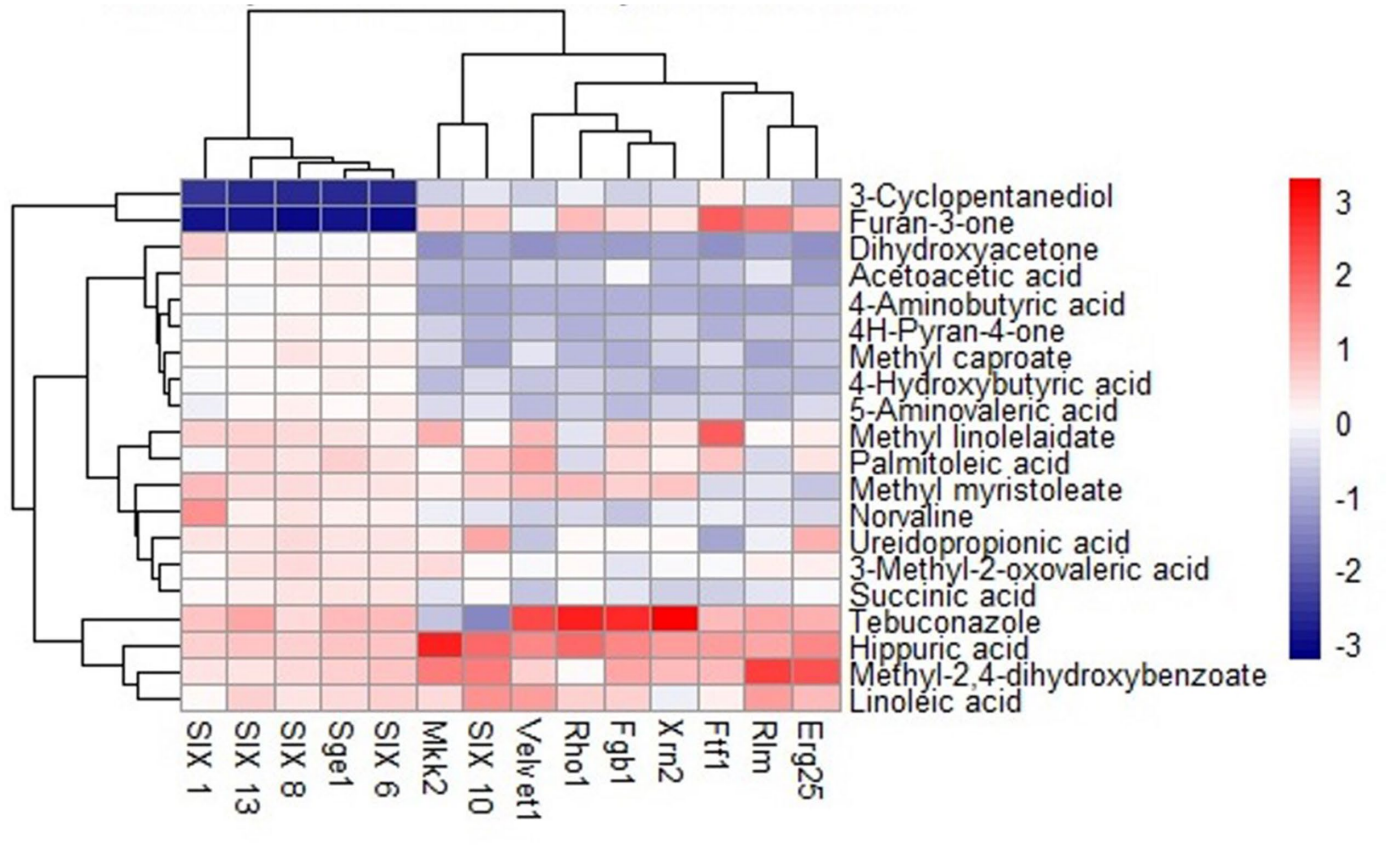
Heat map of molecular docking analysis between *S. thalpophilum* metabolites and protein targets of *Fusarium oxysporum* f. sp. *cubense*.

The outcomes of the docking analysis are depicted in Figure 2, wherein the molecular docking study unveiled the remarkable binding affinity of biomolecules, namely methyl-2,4-dihydroxybenzoate and hippuric acid derived from NMS02 S296, towards the protein targets of *Foc*. These biomolecules exhibited excellent binding affinity (lower binding energies), slightly below that of the positive control, tebuconazole. The lower binding energies signify robust affinities between the compounds and the protein targets, suggesting the potential for enhanced and more efficacious interactions.

**Figure 3:**
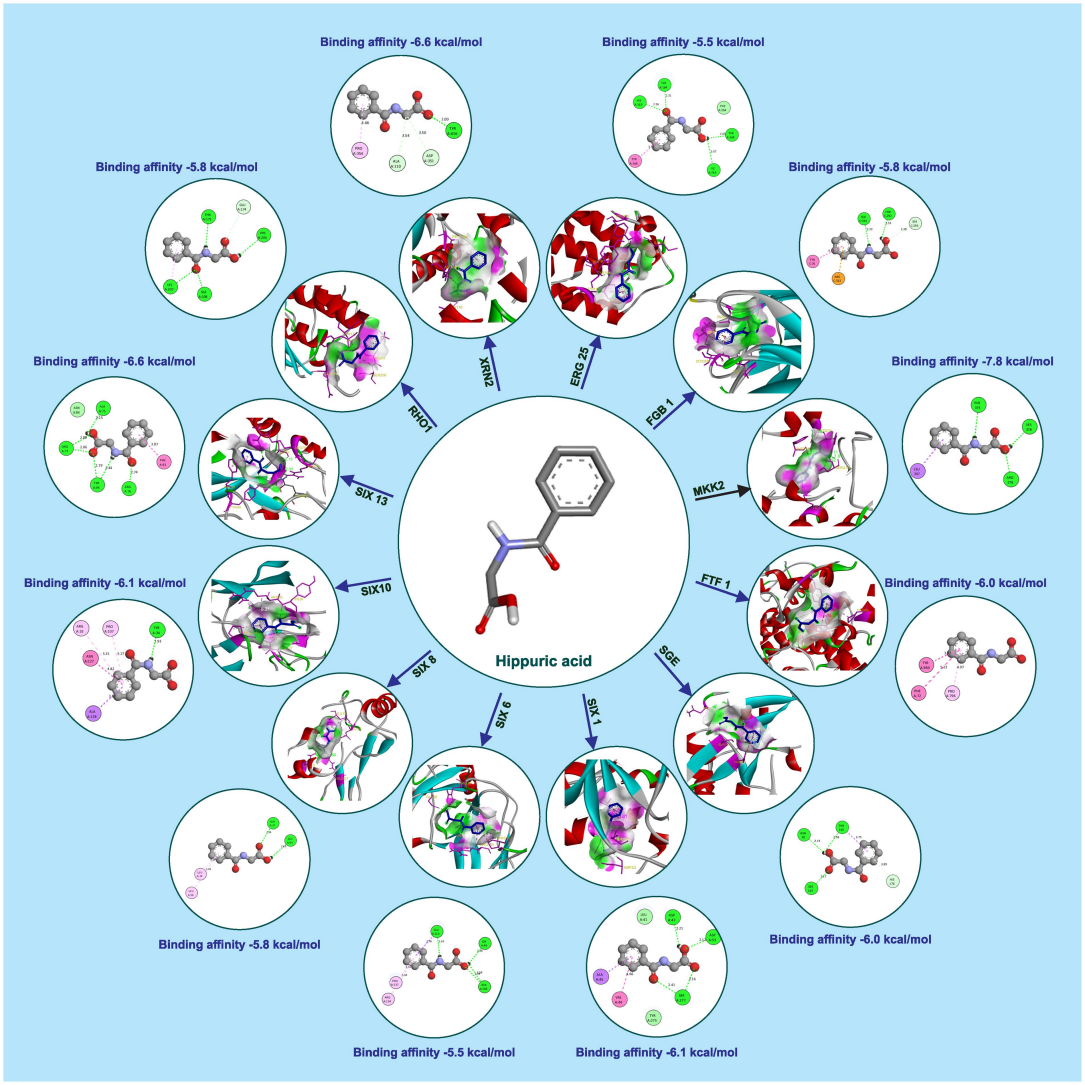
Molecular docking interaction of hippuric acid with active site residues of *Foc* protein targets.

Hippuric acid exhibited a strong binding affinity of −7.8 kcal/mol with Mkk2 (H-bonds; PHE 375, SER 378, ARG 274) which was the highest of all the targets. It showed a good binding affinity of −6.6 kcal/mol with Xrn2 (H-bonds; TRY 616, ALA 110, ASP 351) and SIX 13 proteins (H-bonds; ALA 73, ARG 77, TYR 94, ARG 76). With SIX 1 (ASP 42, ASP 53, SER 277) and SIX 10 (H-bonds; TYR 34), it showed a binding affinity of −6.1 kcal/mol. The observed binding affinities of hippuric acid with Erg25 (H-bonds; ILE 265, TYR 268, TYR 164, HIS 163), Fgb (H-bonds; SER 334, THR 292, SER 293), Ftf, Rho (H-bonds; THR 171, PHE 205, LYS 207, SER 208, GLU 174), Rlm (H-bonds; PRO 199, PRO 205, ASN 203), Sge (H-bonds; THR 165, ASN 61, SER 162, HIS 176), SIX 6 (H-bonds; GLU 213, GLY 48, ASN 166), SIX 8 (H-bonds; GLN 27, GLU 43), and Velvet were −5.5 kcal/mol, −5.8 kcal/mol, −6.0 kcal/mol, −5.8 kcal/mol, −4.0 kcal/mol, −6.0 kcal/mol, −5.5 kcal/mol, −5.8 kcal/mol, and −6.0 kcal/mol, respectively, (Table 1 and Figure 3).

**Table 1:** Interaction details of hippuric acid with protein targets of *Foc*.

S.No	Protein targets	Binding affinity	Hydrogen bonds	Aminoacids involved in H-bonds	Other interactions
1	Erg25	-5.5	4	ILE 265, TYR 268, TYR 164, HIS 163	Van der Waals, Alkyl
2	Ftf1	-6.0	-	-	Van der Waals, Alkyl, Pi-Pi stacked
3	Mkk2	-7.8	3	PHE 375, SER 378, ARG 274	Pi-sigma
4	Rho1	-5.8	5	THR 171, PHE 205, LYS 207, SER 208	Van der Waals
5	Sge	-6.0	4	THR 165, ASN 61, SER 162, HIS 176	Van der Waals
6	SIX1	-6.1	3	ASP 42, ASP 53, SER 277	Pi-Pi stacked, Pi-sigma
7	SIX6	-5.5	3	GLU 213, GLY 48, ASN 166	Alkyl
8	SIX8	-5.8	2	GLN 27, GLU 43	Alkyl
9	SIX10	-6.1	1	TYR 34	Pi-Pi stacked, Pi-sigma, Alkyl
10	SIX13	-6.6	4	ALA 73, ARG 77, TYR 94, ARG 76	Pi stacked
11	FGB 1	-5.8	3	SER 334, THR 292, SER 293	Pi-stacked, Pi-Anion
12	Rlm	-4.0	3	PRO 199, PRO 205, ASN 203	Pi-stacked, Alkyl
13	Velvet	-6.0	-	-	Alkyl
14	Xrn2	-6.6	3	TRY 616, ALA 110, ASP 351	Alkyl

**Figure 4:**
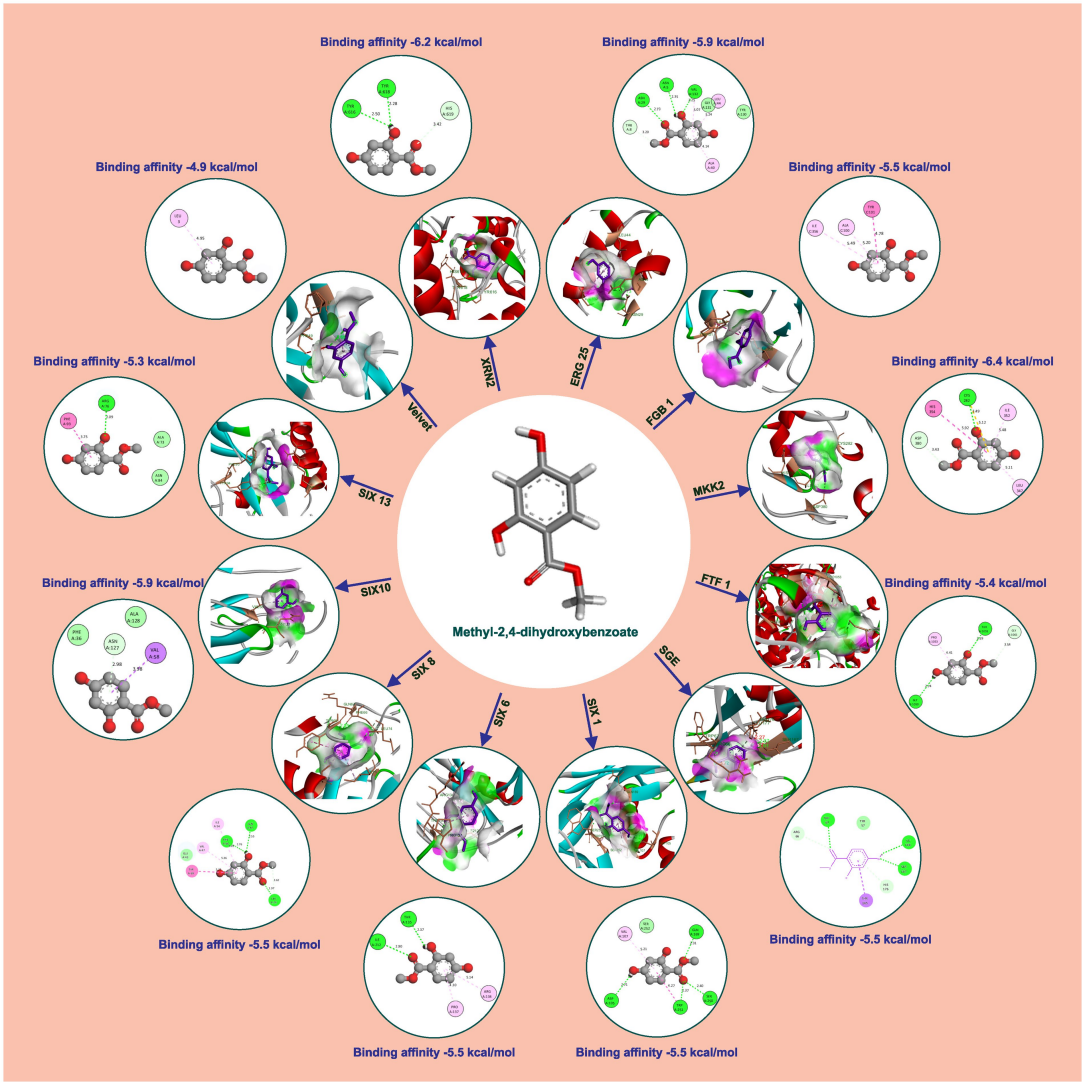
Molecular docking interaction of methyl-2,4-dihydroxybenzoate with active site residues of *Foc* protein targets.

Methyl-2,4-dihydroxybenzoate also exhibits varying degrees of binding affinity with multiple target proteins, each interaction characterized by specific hydrogen bonds. Its binding affinities, measured in kcal/mol, are as follows: −6.4 kcal/mol with Mkk2 (H-bonds; CYS 282), −6.2 kcal/mol with Xrn2 (forming H-bonds with TYR 616, TYR 618), −5.9 kcal/mol with Erg25 (H-bonds; ASN 5, ASN 29, VAL 132) and SIX10, −5.5 kcal/mol with FGB1, Sge (H-bonds; TRP 67, ILE 166, SER 167), SIX1 (H-bonds; ASP 105, GLN 169, SER 250, TRP 251), SIX 6 (H-bonds; ILE 212, THR 135), and SIX 8 (with H-bonds involving PHE 66, GLN 67, LEU 74), −5.4 kcal/mol with Ftf 1 (H-bonds; GLY 1050, THR 1055), −5.3 kcal/mol with SIX 13 (establishing H-bonds with THR 319), −4.9 kcal/mol with Velvet (involving MET 283 and GLN 333), −4.7 kcal/mol with Rlm (H-bonds; ALA 44, GLU 77) and −4.6 kcal/mol with Rho (H-bonds; GLU 110, ARG 293). These distinct binding affinities and the specific residues involved in hydrogen bonding highlight molecules’ diverse interactions with various target proteins, signifying its potential multifaceted biological effects and therapeutic implications (Table 2 and Figure 4).

**Table 2:** Interaction details of methyl-2,4-dihydroxybenzoate with protein targets of *Foc*.

S. No	Protein targets	Binding affinity	Hydrogen bonds	Aminoacids involved in H-bonds	Other interactions
1	Erg25	−5.9	3	Asn 5, Asn 29, Val 132	Van der Waals, Pi - Alkyl
2	Ftf1	−5.4	2	Gly 1,050, Thr 1,055	Pi - Alkyl
3	Mkk2	−6.4	1	Cys 282	Pi – Pi T shaped, Pi - Alkyl
4	Rho1	−4.6	2	Glu 110, Arg 293	Van der Waals, Pi - Alkyl
5	Sge	−5.5	3	Trp 67, Ile 166, Ser 167	Van der aals, Pi - sigma
6	SIX1	−5.5	4	Asp 105, Gln 169, Ser 250, Trp 251	Van der Waals, Pi - Alkyl
7	SIX6	−5.5	2	Ile 212, Thr 135	Pi - Alkyl
8	SIX8	−5.5	3	Phe 66, Gln 67, Leu 74	Van der Waals, Pi-Pi stacked, Pi - Alkyl
9	SIX10	−5.9	–	–	Van der Waals, Pi-Pi sigma
10	SIX13	−5.3	1	Thr 319	Van der Waals, Pi-Pi stacked
11	FGB 1	−5.5	–	–	Pi-Pi stacked, Pi - Alkyl
12	Rlm	−4.7	2	Ala 44, Glu 77	Van der Waals, Pi-Pi stacked, Pi - Alkyl
13	Velvet	−4.9	–	–	Pi-alkyl
14	Xrn2	−6.2	2	Tyr 616, Tyr 618	Van der Waals

The binding affinities of the positive control, tebuconazole, with the different fungal targets were as follows: −8.7 kcal/mol for Xrn2 (H-bonds; ASP A:351, ALA A:110, TYR A:616), −6.8 kcal/mol for Fgb1 (H-bonds; SER C:206, VAL C:294), −6.4 kcal/mol for Rho1 (H-bonds; SER A:137), −6.9 kcal/mol for Velvet, −6.3 kcal/mol for Sge1 (H-bonds; LYS 163), −7.8 kcal/mol for SIX13, −4.0 kcal/mol for Rlm1, −6.8 kcal/mol for SIX1, −5.7 kcal/mol for Ftf1 (H-bonds; CYS A:253, ASP A:110), −4.7 kcal/mol for Mkk2 (H-bonds; ASP A:367), −6.6 kcal/mol for Erg6, −5.2 kcal/mol for Erg25, −5.8 kcal/mol for SIX6 (H-bonds; GLN A:112), −5.3 kcal/mol for SIX8 (H-bonds; TYR A:73), and −4.0 kcal/mol for SIX10 (H-bonds; ALA A:124, ASN A:127).

**Figure 5:**
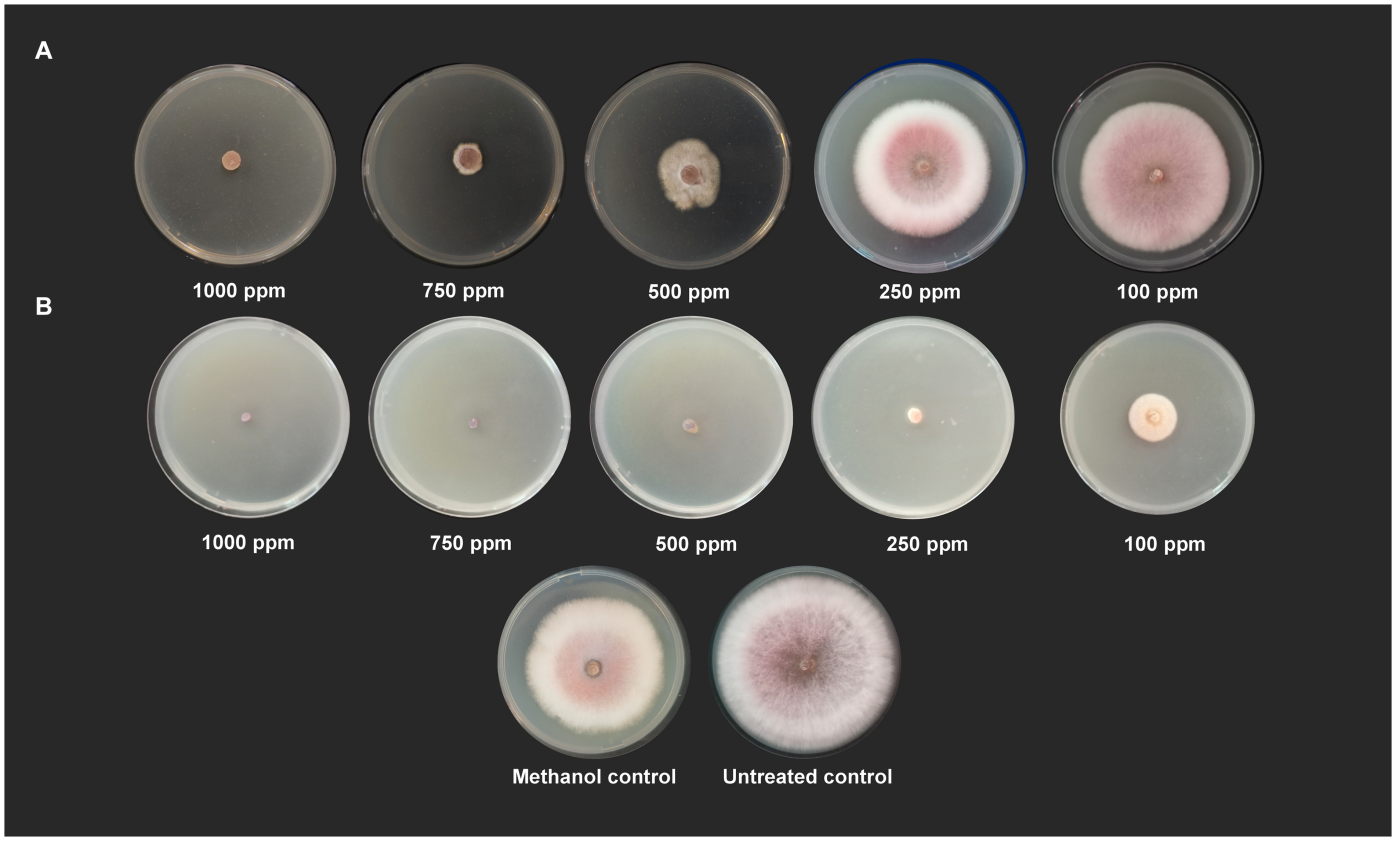
Antifungal activity of **(A)** hippuric acid and **(B)** methyl-2,4-dihydroxybenzoate against *Foc* S16 *in vitro*.

Wet lab testing using poison food technique revealed that hippuric acid and methyl-2,4-dihydroxybenzoate completely halted the growth of *Foc* when tested at a concentration of 1,000 ppm (100% inhibition). Hippuric acid screened plates showed a radial mycelial growth of 7.43 cm, 6.27 cm, 2.87 cm, and 1.52 cm at 100 ppm, 250 ppm, 500 ppm, and 750 ppm, respectively. Methyl-2,4-dihydroxybenzoate showed 100% inhibition at 1000, 750, 500 and 250 ppm. At 100 ppm, radial mycelial growth of 3.16 cm was observed (Figure 5).

### 3.4 Comprehensive genome analysis of *Sphingobacterium thalpophilum* NMS02 S296

Whole genome sequencing yielded 5,824,122 bp (5.8 Mb) with an average G + C content of 43.37%. NMS02 S296 isolate annotated using the RAST tool kit (RASTtk) confirmed it as *Sphingobacterium thalpophilum* and assigned a unique genome identifier of 259.11. The genome belongs to the superkingdom Bacteria and has been annotated using genetic code 11. The genome taxonomy pertains to cellular organisms > Bacteria > FCB group > Bacteroidetes/Chlorobi group > Bacteroidetes > Sphingobacteria > Sphingobacteriales > Sphingobacteriaceae > Sphingobacterium > *Sphingobacterium thalpophilum*. There were 29 contigs in the assembled genome and the shortest contig N50 (sequence length corresponding to 50% of the genome) measured 415,325 bp. In the case of the L50 count, the smallest number of contigs that generated the N50 is 4. In addition, the genome contained 5,341 protein-coding sequences (CDS), 76 transfer RNA (tRNA) genes, and 2 ribosomal RNA (rRNA) genes (Table 3).

**Table 3:** Genome assembly of *S. thalpophilum* NMS02 S296.

Feature	Value
Contigs	29
GC Content	43.37
Contig L50	4
Genome Length (bp)	5,824,122
Contig N50	415,325
CDS	5,341
tRNA	76
rRNA	2

The genome annotation revealed 2,360 hypothetical proteins and 2,981 proteins with assigned functions. Among the proteins with assigned functions, 947 were assigned with Enzyme Commission (EC) numbers, 822 were assigned Gene Ontology (GO) assignments, and 701 proteins were mapped to KEGG pathways. The PATRIC annotation further identified two types of protein families. Specifically, there were 4,507 proteins belonging to the genus-specific protein families (PLFams) and 4,602 proteins belonging to the cross-genus families (PGFams) (Table 4). The annotated genome of *S. thalpophilum* NMS02 S296 is visually represented in a circular graphical format, illustrating the presence of various components such as contigs, coding sequences (CDS), RNA genes, coding sequences associated with known anti-microbial resistance genes, coding sequences associated with virulence factors, GC content, and GC skew (Supplementary Figure S6).

**Table 4:** Protein features of *Sphingobacterium thalpophilum* NMS02 S296.

Hypothetical proteins	2,360
Proteins with functional assignments	2,981
Proteins with EC number assignments	947
Proteins with GO assignments	822
Proteins with pathway assignments	701

Coding sequences of *S. thalpophilum* NMS02 S296 genome for MAMP (Microbe-Associated Molecular Pattern) genes uncovered the presence of translation elongation factor TU (Ef-Tu), which encompasses 1,188 nucleotides and exists between positions 308,559 to 307,372 nucleotides. The genome of NMS02 S296 bacteria exhibited a wide range of genes with various functions. These included seven copies of the peptidoglycan synthetase gene, five genes for glucanase, seven genes for xylanase, one gene for amylase, six genes for glucosidase, one gene for chitin binding protein, two genes for thiol peroxidase, nine genes for phosphate ABC transporter binding protein, and two acetolactate synthase related genes. However, the genome did not possess any arabinase genes, chitosanase-related genes, acetolactate decarboxylase-related genes, and acetoin dehydrogenase-related genes. Further, the genome of *S. thalpophilum* NMS02 S296 comprised specialty genes including antibiotic resistance genes which are 30 in number (Source, PATRIC). Several genes in the annotation show similarity to known transporters, virulence factors, as well as drug targets.

Through the annotation process of *S. thalpophilum* NMS02 S296 genome, an analysis of subsystems was conducted, revealing the presence of superclasses with varying numbers of subsystems (SS) and associated families/genes. This analysis shed light on the genes involved in various biological processes. The genome of NMS02 S296 possessed 68 subsystems, encompassing 483 genes involved in metabolic processes. There were 41 subsystems with 209 genes involved in protein processing, 24 subsystems with 221 genes dedicated to energy-related processes, and 23 subsystems with 82 genes associated with stress response, defense mechanisms, and virulence. Additionally, there were 17 subsystems associated with 77 genes related to DNA processing, and 13 subsystems associated with membrane transport, comprising 262 genes. Furthermore, there were 11 subsystems associated with RNA processing containing 47 genes, 11 subsystems with 58 genes involved in cellular processes, 6 subsystems consisting of 31 genes related to the formation of the cell envelope, and 1 subsystem comprising 3 genes linked to regulation and cell signaling (Supplementary Figure S7).

### 3.5 Comparative genome analysis

The identified ORFs in the isolate NMS02 S296 is 5,103 with a GC content of 44.32%. The genome with the lowest number of ORFs is *Sphingobacterium* sp. GVS05A (NCBI ACC. No. JAHXYR010000010). It encodes 258 ORFs. *S. siyangense* strain T12B17 has the highest number of ORFs totalling 5,736 (Supplementary Figures S8, S9).

**Figure 6:**
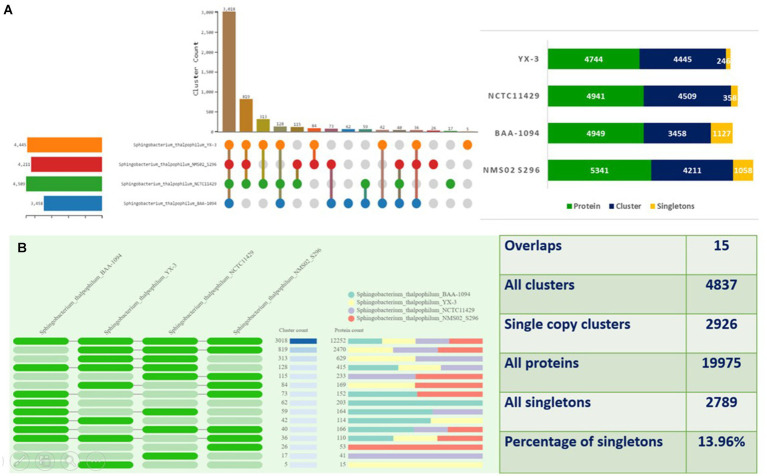
Orthologous cluster analysis of *Sphingobacterium thalpophilum* strains. **(A)** An UpSet plot displaying unique and shared orthologous clusters among the species. The left horizontal bar chart shows the number of orthologous clusters per species, while the right vertical bar chart shows the number of orthologous clusters shared among the species. The lines represent intersecting sets **(B)** overlays identified by the OrthoVenn3 analysis of *Sphingobacterium thalpophilum* strains.

The proteomes of *S. thalpophilum* NMS02 S296 were compared individually with three other *S. thalpophilum* strains (*S. thalpophilum* NCTC11429, *S. thalpophilum* BAA-1094, *S. thalpophilum* YX-3) available in the GenBank database. Orthologous cluster analysis highlights the genetic diversity and potential biocontrol capabilities of *Sphingobacterium thalpophilum* strains. It revealed the presence of 4,837 clusters of orthologous proteins, which include proteins from all four *S. thalpophilum* strains, related by a common ancestral gene. Out of the 4,837 clusters, 2,926 are single-copy clusters, i.e., each cluster containing one protein from each species. There are 2,789 singletons representing 13.96% of all clusters. The 15 overlaps suggested the shared orthologous proteins across these clusters, indicating potential functional similarities or common evolutionary origins. The UpSet plot shows that NMS02 S296 has higher number of unique gene clusters (1,058), suggesting a significant presence of novel genes that could be involved in antifungal activity. The strain also has the highest protein count (5,341) among the strains analyzed, reflecting its diverse metabolic potential (Figure 6).

**Figure 7:**
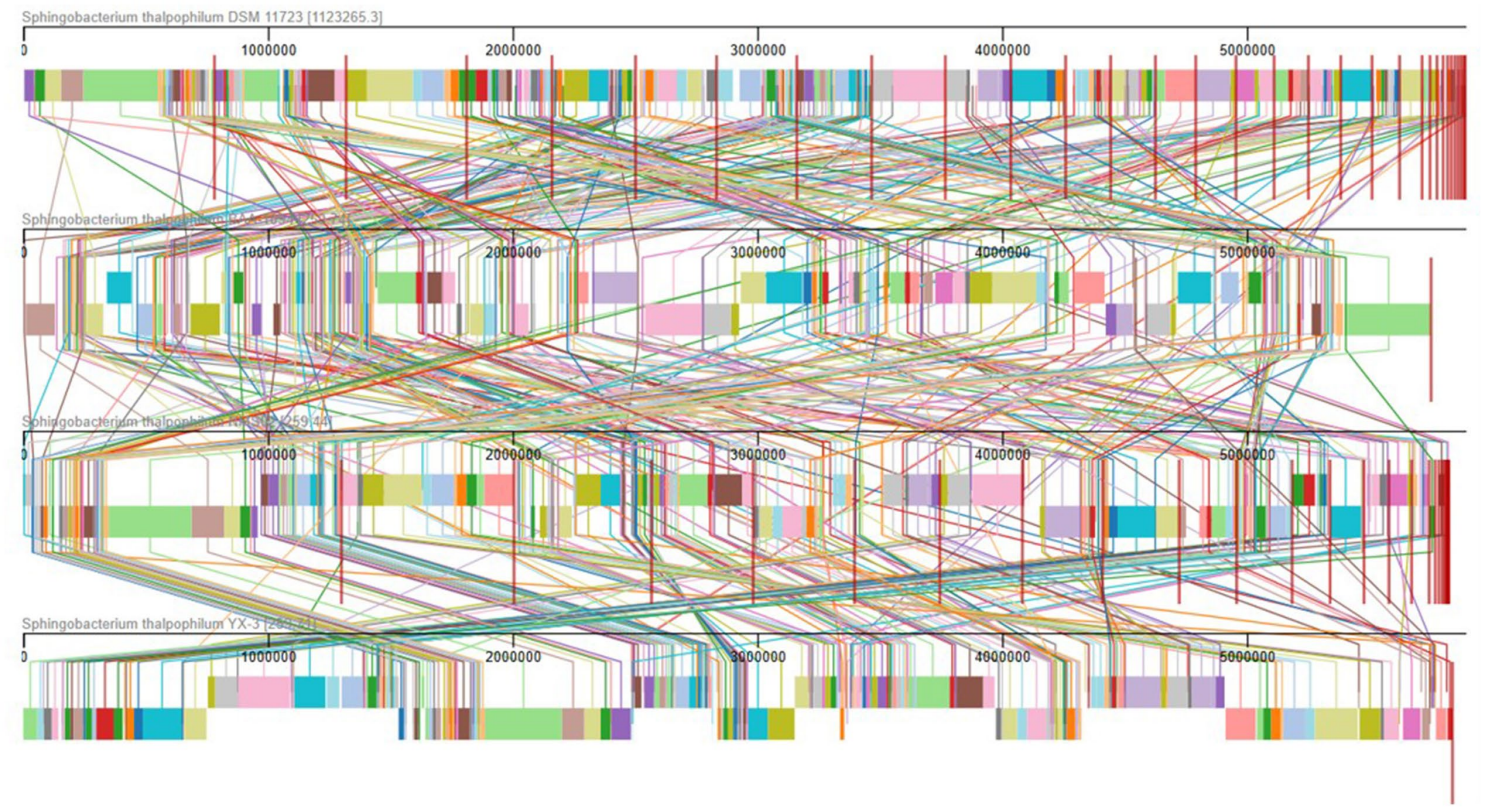
Comparison of NMS02 S296 genome sequences against 4 other *Sphingobacterium thalpophilum* strains using MAUVE. By using MAUVE, pairwise alignments of genomes were generated. Each genome is laid out horizontally with homologous segments outlined as colored rectangles. Rearrangements are shown with colored lines. Boxes with same colour indicate a locally collinear block (LCB) or homologous region (syntenic regions) shared among genomes. The scale is given in nucleotides.

To gain deeper insights into the genomic characteristics of NMS02 S296, a genome synteny analysis was conducted, which involved aligning multiple genomes and comparing them for structural similarities and differences. The analysis, utilizing the MAUVE tool, revealed a significant level of synteny between NMS02 S296 and other *Sphingobacterium thalpophilum* strains (DSM 11723, BAA-1094, and YX-3), indicating a shared genetic foundation and conservation of essential metabolic pathways, stress response mechanisms, and environmental adaptability. The generated synteny map highlighted the presence of local collinear blocks (LCBs) and various regions of translocations, underscoring both conserved and unique genomic regions (Figure 7).

### 3.6 Phylogenetic analysis

The phylogenetic tree (Supplementary Figure S10) illustrates evolutionary lineages over time having a base substitution rate of 0.3 and a bootstrap value (indicating confidence) of 100. Both *S. thalpophilum* NMS02 S296 and *S. thalpophilum* DSM 11723 strain originated from *Sphingobacterium* sp. Ag1, but both do not have any nucleotide substitution at 100% confidence. *S. paucimobilis* HER1398 had more base substitution than the other three *Sphingobacterium* strains. *Pedobacter kyungheensis* KACC 16221 diverged from the *Sphingobacterium* genera with a 0.3 nucleotide substitution rate. The maximum genetic change was observed in the case of *Prevotella stercorea* DSM 18206. The length of the horizontal axis of *Flavobacterium branchiophilum* strain FL-15 from the node indicated that the base substitution rate was higher in *Prevotella stercorea* DSM 18206 when compared to *Riemerella anatipestifer* strains. *Riemerella anatipestifer* ATCC 11845 = DSM 15868 and *Riemerella anatipestifer* ATCC 11845 = DSM 15868 strains did not have any nucleotide substitution at 100% confidence level. The outgroups *Candidatus pelagibacter* strains showed a significant divergence between each other, among which *Candidatus pelagibacter ubique* HIMB083 faced more genetical divergence than the *Candidatus pelagibacter* sp. IMCC9063.

### 3.7 Antimicrobial resistance genes

*Sphingobacterium thalpophilum* NMS02 S296 possesses a diverse repertoire of genes involved in various AMR mechanisms, which potentially contributed to its antimicrobial activity (Table 5). The majority of these shared genes were associated with different mechanisms of antimicrobial resistance. It included genes related to antibiotic activation enzyme (*katG*), antibiotic inactivation enzyme (such as *lnuB*), replacement protein (*fabV*), proteins involved in altering cell wall charge (e.g., *gdpD*), and regulators that modulate the expression of antibiotic resistance genes (*oxyR*).

**Table 5:** Antimicrobial resistance genes in *S. thalpophilum* NMS02 S296.

AMR mechanism	Gene	Encoded Protein/Function
Antibiotic activation enzyme	*katG*	Catalase-peroxidase (EC 1.11.1.21)
Antibiotic inactivation enzyme	*lnuB*	Lincosamide nucleotidyltransferase
Antibiotic target in susceptible species	*ddl*	D-alanine--D-alanine ligase (EC 6.3.2.4)
	*dxr*	1-deoxy-D-xylulose 5-phosphate reductoisomerase (EC 1.1.1.267)
	*ef-G*	Translation elongation factor G
	*ef-Tu*	Translation elongation factor Tu
	*folA*	Dihydrofolate reductase (EC 1.5.1.3)
	*dfr*	Dihydrofolate reductase (EC 1.5.1.3)
	*folP*	Dihydropteroate synthase (EC 2.5.1.15)
	*gyrA*	DNA gyrase subunit A (EC 5.99.1.3)
	*gyrB*	DNA gyrase subunit B (EC 5.99.1.3)
	*iso-tRNA*	Isoleucyl-tRNA synthetase (EC 6.1.1.5)
	*kasA*	3-oxoacyl-[acyl-carrier-protein] synthase, KASII (EC 2.3.1.179)
	*murA*	UDP-N-acetylglucosamine 1-carboxyvinyltransferase (EC 2.5.1.7)
	*rho*	Transcription termination factor
	*rpoB*	DNA-directed RNA polymerase beta subunit (EC 2.7.7.6)
	*rpoC*	DNA-directed RNA polymerase beta subunit (EC 2.7.7.6)
	*S10p*	SSU ribosomal protein S10p (S20e)
	*S12p*	SSU ribosomal protein S12p (S23e)
Antibiotic target replacement protein	*fabV*	Enoyl-[acyl-carrier-protein] reductase (EC 1.3.1.9)
Gene conferring resistance via absence	*gidB*	16S rRNA (guanine(527)-N(7))-methyltransferase (EC 2.1.1.170)
Protein-altering cell wall charge conferring resistance	*gdpD*	Glycerophosphoryl diester phosphodiesterase (EC 3.1.4.46)
Regulator modulating expression of antibiotic resistance	*oxyR*	Hydrogen peroxide-inducible genes activator

### 3.8 Pathways and genes present in the genome of NMS02 S296

Whole Genome Sequencing (WGS) analysis of NMS02 S296 identified various metabolic pathways and different numbers of genes associated with each pathway (Supplementary Table S2). The pathways identified include purine metabolism, arginine, proline metabolism, pyrimidine metabolism, alanine, aspartate, and glutamate metabolism, cysteine and methionine metabolism, fatty acid metabolism, glycine, serine, and threonine metabolism, glycolysis/gluconeogenesis, nitrogen metabolism, pyruvate metabolism, peptidoglycan biosynthesis, amino sugar and nucleotide sugar metabolism, starch and sucrose metabolism, glyoxylate and dicarboxylate metabolism, glycerophospholipid metabolism, selenoamino acid metabolism, tryptophan metabolism, aminoacyl-tRNA biosynthesis, methane metabolism, leucine and isoleucine degradation, phenylalanine, tyrosine, and tryptophan biosynthesis, pentose phosphate pathway, phenylalanine metabolism, biosynthesis of siderophore group non ribosomal peptides, inositol phosphate metabolism, isoquinoline alkaloid biosynthesis, sulfur metabolism, pantothenate and CoA biosynthesis, phenylpropanoid biosynthesis, carotenoid biosynthesis, arachidonic acid metabolism, betalain biosynthesis, flavonoid biosynthesis, puromycin biosynthesis, streptomycin biosynthesis, zeatin biosynthesis, atrazine degradation, and penicillin, cephalosporin biosynthesis, and lipoic acid metabolism pathways.

### 3.9 Regions coding for secondary metabolites

Six distinct regions were identified in the genome of NMS02 S296 that code for secondary metabolites. These regions correspond to contigs 1, 6, 7, 11, 13, and 14 of the genome and are predicted to produce betalactone, hglE-KS/T1PKS, RiPP-like, terpene, and RRE secondary metabolites, respectively. Region 1 is a beta lactone type member and was located between nucleotide positions 475,951 and 501,742 encompassing a total length of 25,791 nucleotides (Supplementary Figure S11). Region 2, belonging to the hglE-KS, T1PKS type member, stretches from nucleotide positions 201,829 to 253,063 (Supplementary Figure S12). Region 3, spanning nucleotide positions ranging from 312,749 to 324,911. It was similar to a RiPP-like type member. No known clusters exhibited similarity to these three regions (Supplementary Figure S13). Region 4, ranged from nucleotide positions 1840 to 22,676. It was similar to a terpene-type member. It bears a resemblance of 28% to the carotenoid cluster (Supplementary Figure S14). Region 5, of the RRE-containing type member spans from position 120,442 to 138,552 nucleotide. This region does not have similarities to any known clusters (Supplementary Figure S15). Further, region 6 (Supplementary Figure S16) corresponds to a saccharide cluster known as S-layer glycan with 20% similarity. It spans nucleotide positions ranging from 30,065 to 50,337. It was about RRE-containing type members (Table 6).

**Table 6:** Secondary metabolite regions identified in the genome of *Sphingobacterium thalpophilum* NMS02 S296.

Region	Type	Nucleotide		Most similar known cluster	Similarity%	Most similar microbial species
		From	To			
Region 1	betalactone	475,951	501,742	–	–	*S. thalpophilum* strain NCTC11429
Region 2	hglE-KS, T1PKS	201,829	253,063	–	–	–
Region 3	RiPP-like	312,749	324,911	–	–	–
Region 4	terpene	1,840	22,676	Carotenoid	28%	*Sphingobacterium multivorum*
Region 5	RRE-containing	120,442	138,522	–	–	–
Region 6	RRE-containing	30,065	50,337	S-layer glycan	20%	*Flavobacterium johnsoniae*

hglE-KS, heterocyst glycolipid synthase-like polyketide synthase; T1PKS, Type I polyketide synthase; RiPP, ribosomally synthesized and post-translationally modified peptide; RRE, RiPP recognition elements.

**Figure 8:**
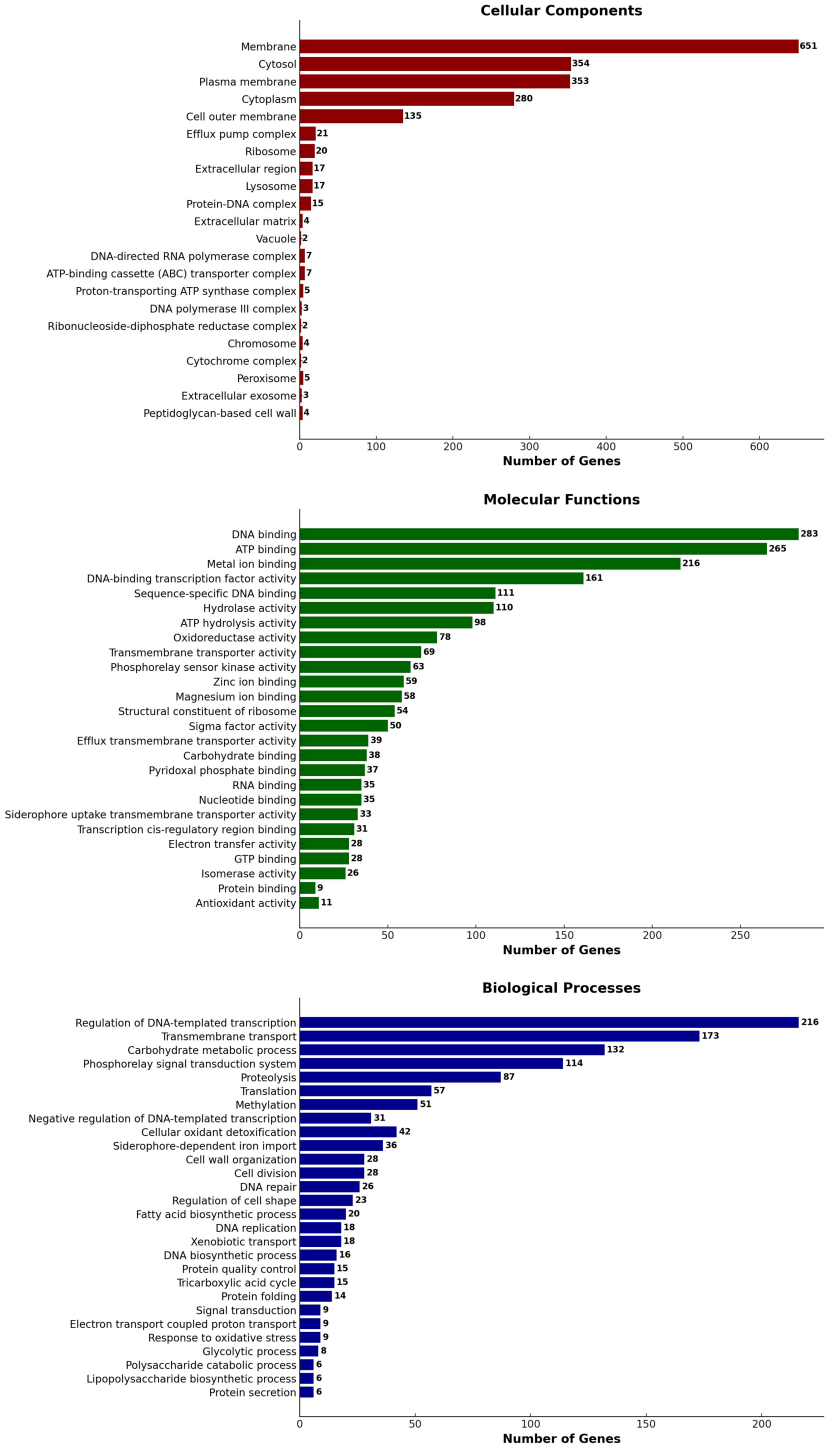
Gene ontology (GO) annotation and functional classification of *S. thalpophilum* NMS02 S296 genome.

Using Blast 2 GO annotation in OmicsBox 3.2.4, a functional categorization by gene ontology (GO) terms was conducted. The analysis was based on the Blastx hits from the non-redundant database. In total, 28 GO terms related to biological processes, 22 GO terms related to cellular components, and 26 GO terms related to molecular function classes were identified (Figure 8). The Gene Ontology (GO) annotation and functional classification of *S. thalpophilum* NMS02 S296 reveal a robust potential for biocontrol and antifungal activities. A significant number of genes are associated with membrane structures, cytosol, and plasma membrane, indicating a strong capability for interaction with the environment and secretion of bioactive compounds. The presence of genes related to efflux pump complexes and extracellular regions suggests mechanisms for secreting antifungal agents. Molecular functions such as DNA binding, ATP binding, and metal ion binding play critical roles in gene regulation and enzyme activity, facilitating the production of antifungal metabolites. Additionally, hydrolase and oxidoreductase activities point to the organism’s ability to degrade fungal cell walls and manage oxidative stress, enhancing its antifungal efficacy. Biological processes such as the regulation of DNA-templated transcription, transmembrane transport, and carbohydrate metabolic processes underscore the organism’s capability to synthesize and transport antifungal compounds efficiently. The involvement in proteolysis, translation, and methylation further supports the production of a diverse array of bioactive molecules. The genes involved in cell wall organization and repair suggest that *S. thalpophilum* NMS02 S296 can maintain its structural integrity while producing antifungal substances. Overall, the GO annotation and functional classification highlight the organism’s sophisticated system for producing, secreting, and managing antifungal agents, making it a promising candidate for biocontrol applications.

### 3.10 Pan-genome analysis

**Figure 9:**
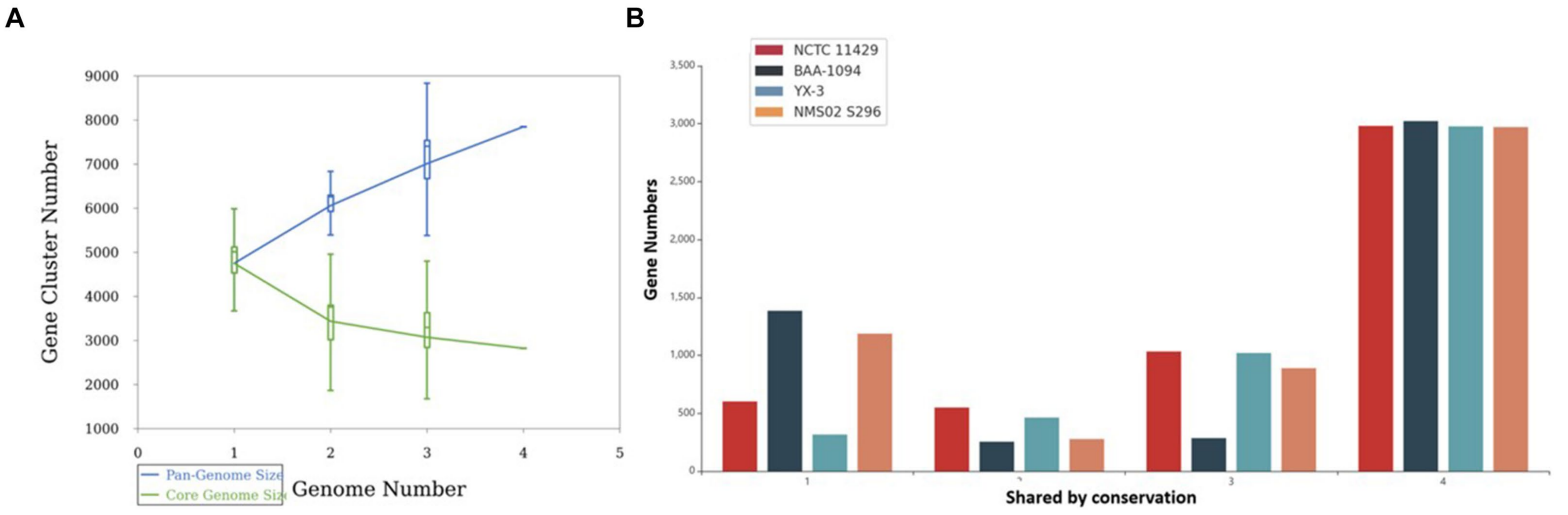
**(A)** Gene accumulation curves of the pan-genome (blue) and core-genome (green) of 4 Sphingobacterium thalpophilum strains instead of Gene accumulation curves of the pan-genome (blue) and core-genome (green) of 8 Sphingobacterium spp. The blue boxes denote each pan-genome size of each genome for comparison. The green boxes show the *Sphingobacterium* core genome size of each genome for comparison. **(B)** Gene distribution by conservation in *S. thalpophilum* strains.

Pan-genome analysis showcased the variability in gene presence among different strains of *Sphingobacterium thalpophilum*. It revealed a dynamic genome structure characterized by a decreasing core genome and an expanding pan-genome. The pan-genome is represented as a blue curve, illustrating the total gene repertoire across four *S. thalpophilum* strains. The core genome is depicted as a green curve, signifying the set of genes shared among all four strains. As the number of genomes analyzed increases from one to four, the pan-genome size exhibits a continuous increase, starting from approximately 4,500–5,000 gene clusters with one genome to about 7,000–8,000 gene clusters with four genomes. This indicates that each additional genome contributes unique genes, although the rate of new gene discovery decreases over time. Concurrently, the core genome size, which represents genes shared by all strains, shows a decreasing trend, dropping from around 4,500 gene clusters with a single genome to a stable range of 3,000–3,500 gene clusters when four genomes are considered (Figure 9A). This decline highlights the genetic diversity among the strains, as fewer genes are universally shared. The core genome size stabilizes after including multiple genomes, suggesting a consistent set of essential genes common to all strains.

In terms of gene distribution by conservation, the core genome, shared by all four strains, includes 2,971–3,023 genes. Specifically, NCTC11429 shares 2,981 genes, BAA-1094 shares 3,023 genes, YX-3 shares 2,977 genes, and NMS02 S296 shares 2,971 genes. Genes shared by any three strains range from 285 to 1,033, with NCTC11429 sharing 1,033 genes, BAA-1094 sharing 285 genes, YX-3 sharing 1,020 genes, and NMS02 S296 sharing 889 genes. For genes shared by any two strains, the numbers range from 254 to 549, with NCTC11429 sharing 549 genes, BAA-1094 sharing 254 genes, YX-3 sharing 463 genes, and NMS02 S296 sharing 277 genes. Unique genes, shared by only one strain, range from 316 to 1,385, with NCTC11429 having 602 unique genes, BAA-1094 having 1,385, YX-3 having 316, and NMS02 S296 having 1,187 unique genes. These results collectively underscore the genetic diversity and adaptability of *Sphingobacterium thalpophilum* while identifying a stable core genome essential for basic functions (Supplementary Table S3 and Figure 9B).

Among the four strains of *Sphingobacterium thalpophilum*, key biocontrol-associated genes were compared. It was observed that *S. thalpophilum* NMS02 S296 strain possesses the highest number of glucosidase (6 Nos.) and xylanase (7 Nos.) genes, indicating a strong capability to degrade glucosides and xylans, which are key components of pathogen cell walls. Additionally, with 6 glucanase genes, NMS02 S296 shows considerable potential in degrading glucans. Notably, chitin-binding protein genes were present in NMS02 S296 but absent in the other strains compared. The study strain also has genes for thiol peroxidase (2 No.) and acetolactate synthase (2 No.), suggesting a good capacity for oxidative stress protection and branched-chain amino acid biosynthesis, respectively. Despite having fewer amylase (1 No.) and chitin-binding protein (1 No.) genes, *S. thalpophilum* NMS02 S296’s overall enzyme profile highlights its efficacy in biocontrol. Specifically, in our study strain NMS02 S296, we identified a total of 15 MAMP genes, with 8 genes associated with elongation factor and 7 genes related to peptidoglycan. The highest cumulative count of these genes was observed in NMS02 S296 when compared to other *Sphingobacterium* strains (Supplementary Figure S17).

The phylogenetic tree constructed using the pan-based neighbour-joining method elucidates the evolutionary relationships among four *Sphingobacterium thalpophilum* strains: YX-3, BAA-1094, NMS02 S296, and NCTC11429. The tree structure reveals that NCTC11429 is the most divergent strain, branching off earliest from the common ancestor. The remaining three strains form a distinct clade, indicating closer genetic relationships among them. Within this group, YX-3 appears slightly more distant, while BAA-1094 and NMS02 S296 show the closest relationship, sharing a more recent common ancestor. This arrangement suggests a gradual divergence pattern within the species, with NCTC11429 potentially representing an earlier evolutionary branch. The relatively similar branch lengths imply comparable levels of genetic divergence between the strains (Supplementary Figure S18).

### 3.11 Mode of action of *Sphingobacterium thalpophilum* NMS02 S296 against *Foc*


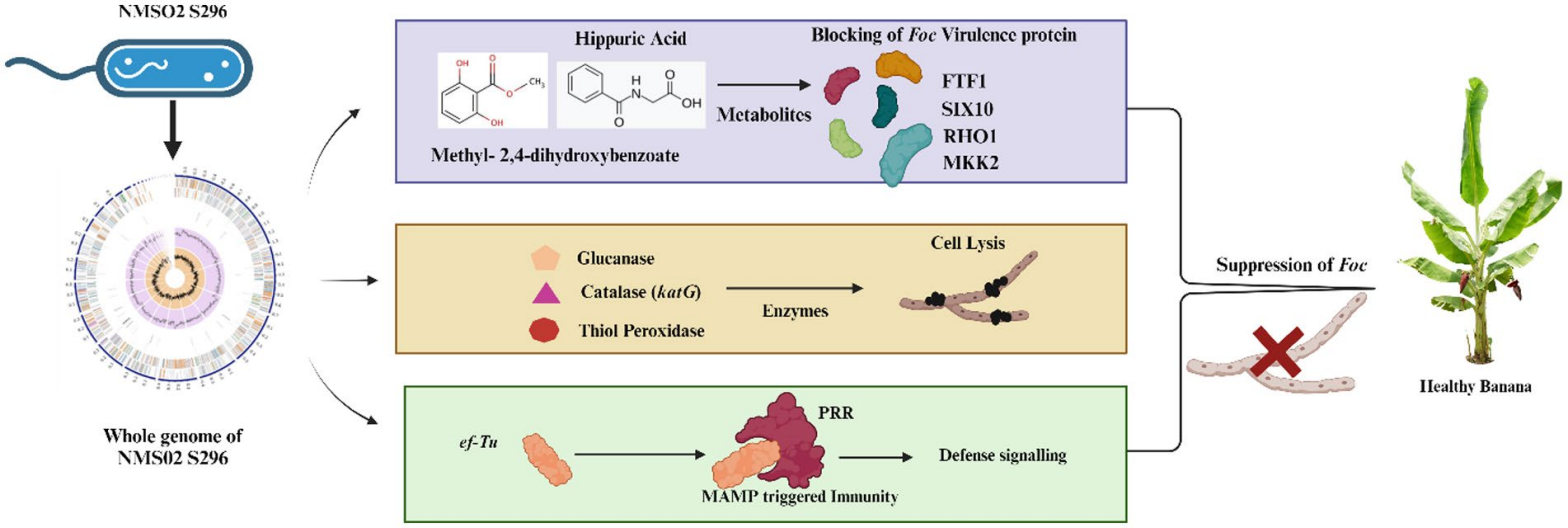


## 4 Discussion

In the present investigation, we have assessed 16 bacterial antagonists for their antifungal activity against *Foc*, the devastating pathogen causing Fusarium wilt in banana plants. *S. thalpophilum* NMS02 S296 isolated from the resistant banana cultivar *Pisang lilin* exhibited exceptional antifungal potential during the confrontational assay when compared to the other bacterial endophytes. However, research on the exploration of the antagonistic behaviour of *Sphingobacterium thalpophilum* has been rarely explored to suppress fungal pathogens. There are only a few WGS records available for *S. thalpophilum* in the NCBI database. Therefore, a comprehensive understanding of the genes present in the genome of the test strain is imperative to fish out the organism’s full potential (Narayanasamy et al., 2023). Considering this research gap, a comprehensive analysis of the complete genome sequence of NMS02 S296 was conducted to ensure precise taxonomic classification and delineate genetic elements or metabolic pathways that could potentially be linked to its capability in hindering fungal growth and exhibiting antifungal properties. When comparing the test isolate NMS02 S296 with the genomes of other isolates and closely related species, notable variations were observed in the encoded Open Reading Frames (ORFs). These variations might emanate from a heightened incidence of horizontal gene transfer or potential gaps in available genomic data. Consequently, this discrepancy offered an opportunity for future investigations that aided in unravelling the origins of differences in ORF counts across these genomes. One of the remarkable features of *S. thalpophilum* NMS02 S296 refers to the diverse genetic repository, encoding for antimicrobial compounds and different metabolic pathways. Comparative genomic analyses with other biocontrol agents have revealed unique clusters of genes within *S. thalpophilum* NMS02 S296 associated with the biosynthesis of specialized secondary metabolites. Interestingly, the presence of distinct hglE-KS/T1PKS (heterocyst glycolipid synthase-like polyketide synthase/Type I polyketide synthase) and RiPPs (Ribosomally synthesized and post-translationally modified peptides) in its genome reveals the production of potent biomolecules contributing towards the antifungal activity against *Fusarium* species. Hitherto, the specificity and novelty of these biosynthetic gene clusters in *S. thalpophilum* NMS02 S296 reveal the potential of this organism to be explored as a promising candidate for biological control of fungal pathogens.

The whole genome sequence of *S. psychroaquaticum* strain SJ-25 revealed the presence of numerous functional genes responsible for antibiotic resistance, the production of siderophores, chitinase, and various antimicrobial compounds (Xu et al., 2020). Similarly, in-depth genome analysis of *S. thalpophilum* NMS02 S296 revealed an overabundance of genes related to hydrolytic enzymes, biosynthesis of siderophores, phytohormones, and antimicrobial resistance genes. The presence of amylases, xylanases, glucanases, glucosidases, and chitin-binding protein genes in the genome of *S. thalpophilum* NMS02 S296 might contribute to the hyper parasitic mechanism and lysis of the fungal pathogen. Thus, the array of enzymatic arsenal in NMS02 S296 bestows the organism with an added advantage over other biocontrol agents by facilitating it with multiple modes of action against the pathogen. It might also contribute towards the effective suppression of *Foc*. The presence of chitin-binding protein genes in NMS02 S296 might enable it to interact effectively with chitin present in the fungal cell wall. The thiol peroxidase genes in NMS02 S296 might be also responsible for the antifungal action by protecting the plant from oxidative stress induced by fungal pathogens and by regulating the redox signalling pathways in plants involved in defense responses against fungal pathogens. Our study isolate NMS02 S296 has acetolactate synthase (ALS) genes responsible for acetolactate synthase (ALS) production. This enzyme is crucial for synthesizing acetoin, a volatile organic compound (VOC) known to significantly enhance plant growth promotion. Likewise, ALS genes were observed in *B. licheniformis* GL174, a culturable endophyte of *Vitis vinifera* cv. Glera demonstrated its involvement in fostering growth promotion (Nigris et al., 2018). Similarly, the research by Saravanan et al. (2021) unveiled that the genome of *B. velezensis* VB7 (CP047587) was characterized with MAMP genes including elongation factor, flagellin and non-ribosomal peptide synthetase gene clusters associated responsible for the antifungal activity against *Foc*.

The comparative analysis of the *S. thalpophilum* NMS02 S296 genome with 4 other strains revealed a widespread distribution of these strains originating from various countries, including USA and China. However, based on the available literature and to the best of our knowledge, this is the first work that depicts the whole genome analysis of NMS02 S296 in India. To our surprise, the genome of NMS02 S296 exhibited a higher potential compared to other *Sphingobacterium thalpophilum* strains reported from global locations. The pangenome analysis of *Sphingobacterium thalpophilum* NMS02 S296, alongside other strains, reveals significant insights into the genetic diversity and adaptive potential of this species. Our findings align with those of recent studies, such as Jia and Lu (2024), who conducted a comprehensive pangenome analysis of nine endophytic strains and thirty-three type strains of the genus *Burkholderia*. They discovered that *Burkholderia* possesses an open pangenome, highlighting its extensive adaptive capacity. Similarly, the open nature of the *S. thalpophilum* pangenome suggests a high degree of genetic variability and adaptability, which is crucial for its role as a biocontrol agent and its interactions within the plant microbiome. Kadiri et al. (2023) revealed the biocontrol capabilities of *B. velezensis* VB7 against *Phytophthora infestans* through pan-genome analysis and molecular docking. The extensive repertoire of heat shock proteins suggests that the study isolate NMS02 S296 possesses robust mechanisms for protein folding, assembly, and stress response. The presence of multiple heat shock proteins may enhance their ability to thrive under various conditions, including those encountered during biocontrol applications. These proteins can aid in the proper folding and stability of proteins involved in interactions with plant hosts or antagonistic activities against pathogens.

*In vitro*, assays conducted in this study witnessed the robust antagonistic activity of *S. thalpophilum* NMS02 S296 against Panama wilt pathogen *Foc*. Comparative studies with conventional biocontrol agents have also demonstrated that *S. thalpophilum* NMS02 S296 was superior in its antifungal action against *Foc* as evidenced by larger inhibition zones and more pronounced suppression of mycelial growth. Such heightened efficacy suggested that *S. thalpophilum* NMS02 S296 might have offered a more potent and targeted approach in combatting Fusarium wilt compared to other biocontrol agents. A bacterial endophyte, *B. amyloliquefaciens* NJN-6W19 produced various antifungal compounds, including lipopeptides and VOCs responsible for the antifungal action of soil-borne pathogens (Wang et al., 2013; Ashburner et al., 2000). In this study, the biomolecules generated by *S. thalpophilum* NMS02 S296 were identified as 2-methyl caproate, 1,3-cyclopentanediol, dihydroxy dimethyl furanone, 3-methyl-2-oxovaleric acid, 5-aminovaleric acid, 4H-pyranone, dihydroxyacetone, norvaline, acetoacetic acid, aminobutyric acid, succinic acid, hippuric acid, methyl-2,4-dihydroxybenzoate, methyl linolelaidate, ureidopropionic acid, 4-hydroxybutyric acid, palmitoleic acid, linoleic acid, and methyl myristoleate. Wet lab experimentation using the poisoned plate technique confirmed the antifungal properties of hippuric acid and methyl-2,4-dihydroxybenzoate. Results demonstrated its complete inhibition of *Foc* growth at a concentration of 1,000 ppm. Maung et al. (2021) documented the inhibitory impact of methyl hippurate on both spore germination and mycelial growth of *B. cinerea*. Chen et al. (2020) highlighted the broad-spectrum antifungal efficacy of methyl benzoate, emphasizing its potential as a promising candidate for the development of bio fungicides. Despite these advancements, the translation of laboratory findings to field applications warrants comprehensive assessments. Future studies will also explore the interactions of *S. thalpophilum* NMS02 S296 with indigenous microbial communities and evaluate its performance under diverse environmental conditions, including different soil types and climatic variations, to ascertain its robustness and efficacy as a biocontrol agent in practical agricultural settings.

## 5 Conclusion

The assessment of various bacterial endophytes for their antagonistic effects against *Foc* revealed diverse levels of antifungal activity. Among the tested isolates, *S. thalpophilum* NMS02 S296 emerged as notably potent, exhibiting a remarkable inhibition percentage of 57.88% against *Foc*. This isolate showcased significantly higher antifungal efficacy compared to earlier isolates previously identified as effective against *Foc*. To gain deeper insights into the mechanisms underlying its potent antifungal activity, additional investigations were conducted. Gas Chromatography–Mass Spectrometry (GCMS) analysis was employed to identify and characterize the specific bioactive compounds present within *S. thalpophilum* NMS02 S296. The GCMS analysis identified several bioactive compounds within the isolate.

Furthermore, molecular docking studies were performed to elucidate the potential interactions between the bioactive compounds identified through GC–MS analysis and the target components within *Foc*. Molecular Docking serves as a computational tool to predict the binding affinities and interactions between molecules, providing insights into the possible modes of action of these bioactive compounds against *Foc*. The results of the molecular docking studies offered a theoretical framework, highlighting potential molecular mechanisms underlying the inhibitory effects of *S. thalpophilum* NMS02 S296 against *Foc*. Experimental validation in the wet lab utilizing the poisoned food technique affirmed the antifungal efficacy of both bioactive compounds viz., hippuric acid and methyl-2,4-dihydroxybenzoate. The comprehensive genomic analysis and *in vitro* assays presented herein underscore the exceptional and distinctive antifungal potential of *S. thalpophilum* NMS02 S296 as a biocontrol agent against *Foc*. Its unique genomic features and broad spectrum of action against Panama wilt pathogen and the presence of unique genes of different nature would aid to explore *S. thalpophilum* NMS02 S296 as a promising candidate for further development in sustainable agricultural practices and highlight avenues for exploring its distinct mechanisms of action for mitigating the looming threat imposed by *Fusarium oxysporum* f.sp. *cubense*.

## Data Availability

The datasets presented in this study can be found in online repositories. The names of the repository/repositories and accession number(s) can be found in the article/Supplementary material.
