# Supplementary material for: Insights on mining the pangenome of Sphingobacterium thalpophilum NMS02 S296 from the resistant banana cultivar Pisang lilin confirms the antifungal action against Fusarium oxysporum f. sp. cubense
Source: Front Microbiol. 2024 Sep 19;15:1443195. doi: 10.3389/fmicb.2024.1443195 (PMC11446778; doi:10.3389/fmicb.2024.1443195)
Supplement: Supplementary file 1 [file Data_Sheet_1.docx]

**Table S1.** **List of bacterial endophytes received from TNAU culture collection center**

| **SL. No:** | **Bacterial endophytes** | **Accession Number** |
| --- | --- | --- |
| 1 | *Bacillus atrophaeus* CNEB8 | MZ485377 |
| 2 | *Serratia rubidaea* YEB PT3 | MN056893 |
| 3 | *Bacillus subtilis* YEB L5 | MK259031 |
| 4 | *Bacillus mojavensis* CNEB14 | MZ485478 |
| 5 | *Bacillus albus* YKMN2 | MT120179 |
| 6 | *Bacillus siamenis* YKMN1 | MT119641 |
| 7 | *Bacillus glycinifermentans* CNEB17 | MZ485782 |
| 8 | *Bacillus velezensis* YEBFL3 | MT326231 |
| 9 | *Bacillus altitudinans* YEBFR3 | MT326232 |
| 10 | *Bacillus barbaricus* NPBR1 | MT383635 |
| 11 | *Bacillus tequelensis* YEB PS1 | MT326211 |
| 12 | *Bacillus sonorensis* KM ROOT 3 | MW331689 |
| 13 | *Bacillus licheniformis* YEBFL5 | MT326233 |
| 14 | *Bacillus amyloliquefaciens* CNEB24 | MZ485912 |
| 15 | *Bacillus megatherium* YEBFL4 | MT326235 |
| 16 | *Sphingobacterium thalpophilum* NMS02 S296 | JALHSB01 |

**Table S2. List of annotated pathways of *S. thalpophilum* NMS02 S296**

| **Pathway name** | **Number of genes** |
| --- | --- |
| Purine metabolism pathway | 37 |
| Arginine and proline metabolism | 19 |
| Pyrimidine metabolism | 27 |
| Alanine, aspartate and glutamate metabolism | 10 |
| Cysteine and methionine metabolism | 14 |
| Fatty acid metabolism | 7 |
| Glycine, serine and threonine metabolism | 16 |
| Glycolysis / gluconeogenesis | 11 |
| Nitrogen metabolism | 32 |
| Pyruvate metabolism | 11 |
| Peptidoglycan biosynthesis | 10 |
| Amino sugar and nucleotide sugar metabolism | 21 |
| Starch and sucrose metabolism | 18 |
| Glyoxylate and dicarboxylate metabolism | 11 |
| Glycerophospholipid metabolism | 16 |
| Selenoamino acid metabolism | 7 |
| Tryptophan metabolism | 11 |
| Aminoacyl-tRNA biosynthesis | 21 |
| Sphingolipid metabolism | 21 |
| Methane metabolism | 6 |
| Valine, leucine and isoleucine degradation | 9 |
| Phenylalanine, tyrosine and tryptophan biosynthesis | 18 |
| Pentose phosphate pathway | 13 |
| Phenylalanine metabolism | 3 |
| Biosynthesis of siderophore group nonribosomal peptides | 4 |
| Inositol phosphate metabolism | 4 |
| Isoquinoline alkaloid biosynthesis | 1 |
| Sulfur metabolism | 2 |
| Pantothenate and CoA biosynthesis | 10 |
| Phenylpropanoid biosynthesis | 1 |
| Carotenoid biosynthesis | 1 |
| Arachidonic acid metabolism | 1 |
| Flavonoid biosynthesis | 1 |
| Puromycin biosynthesis | 1 |
| Streptomycin biosynthesis | 9 |
| Zeatin biosynthesis | 2 |
| Atrazine degradation | 3 |
| Penicillin and cephalosporin biosynthesis | 1 |
| Lipoic acid metabolism | 2 |

**Table S3. Distribution of genes in *Sphingobacterium thalpophilum* strains**

| **No. of Strains Sharing Genes** | **NCTC 11429 (Gene Count)** | **BAA-1094 (Gene Count)** | **YX-3**  **(Gene Count)** | **NMS02 S296 (Gene Count)** |
| --- | --- | --- | --- | --- |
| **4 (Core genome)** | 2,981 | 3,023 | 2,977 | 2,971 |
| **3** | 1,033 | 285 | 1,020 | 889 |
| **2** | 549 | 254 | 463 | 277 |
| **1 (Unique genes)** | 602 | 1,385 | 316 | 1,187 |


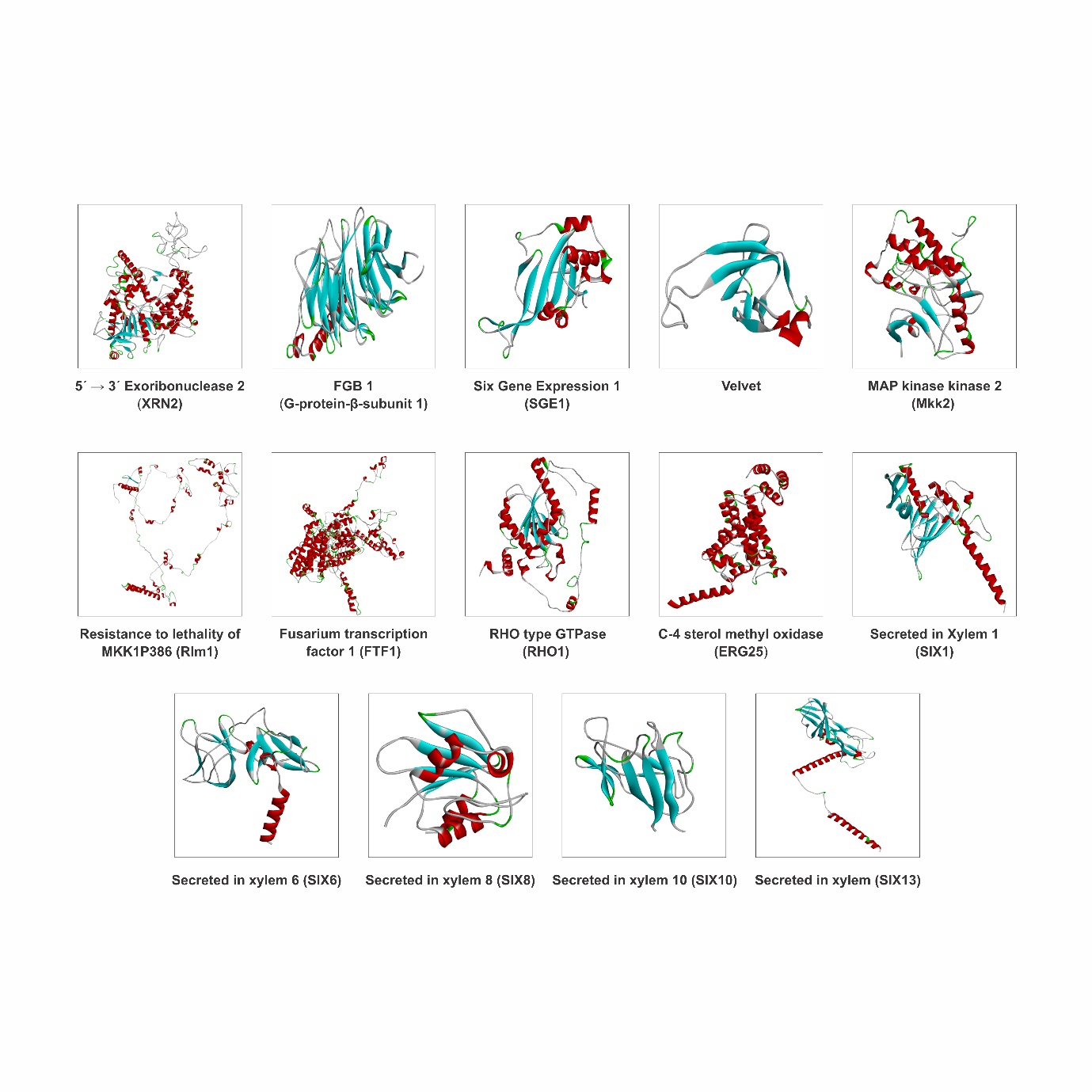


**Fig S1.** Three-dimensional protein structures linked to the virulence and pathogenic traits of *Foc*


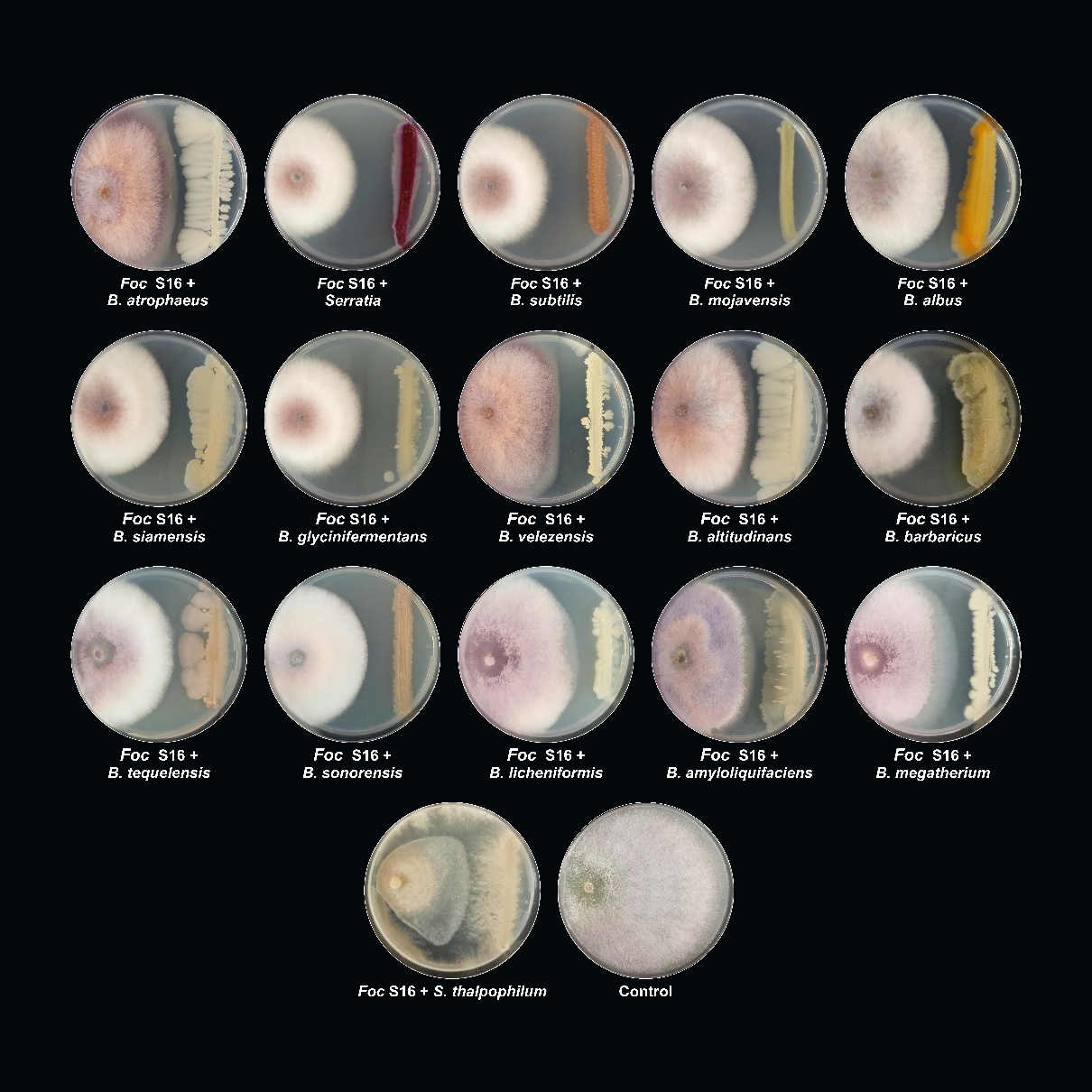


**Fig S2.** *In vitro* assessment of antifungal activity of bacterial endophytes against *Foc*


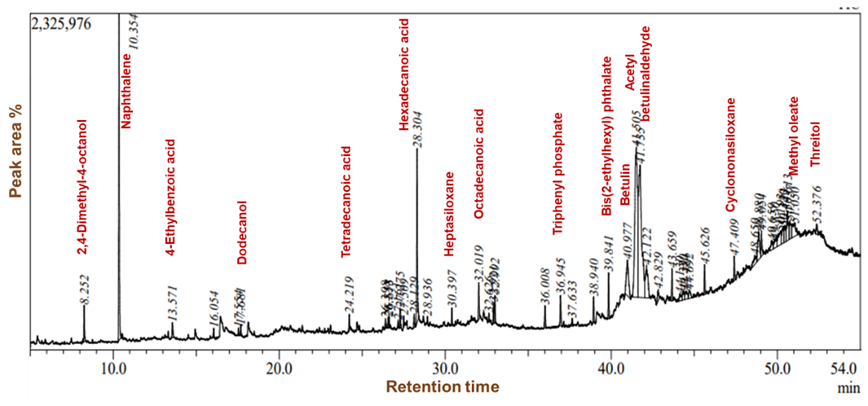


**Fig S3.** GC-MS chromatogram of VOCs/NVOCs produced by *Foc* alone


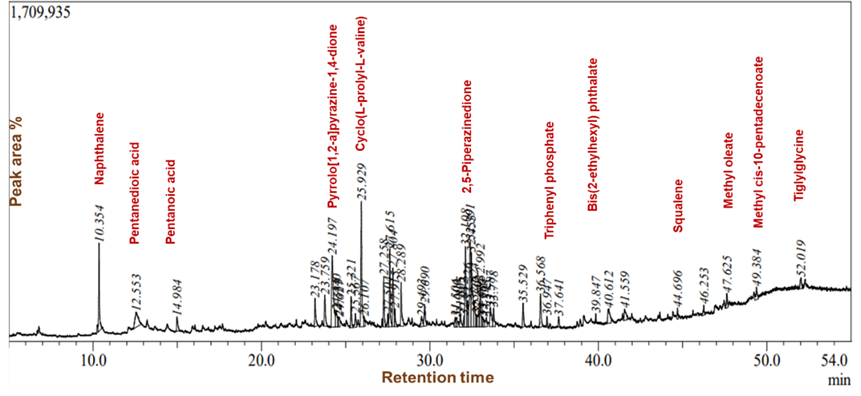


**Fig S4.** GC-MS chromatogram of VOCs/NVOCs produced by *S. thalpophilum* NMS02 S296 alone


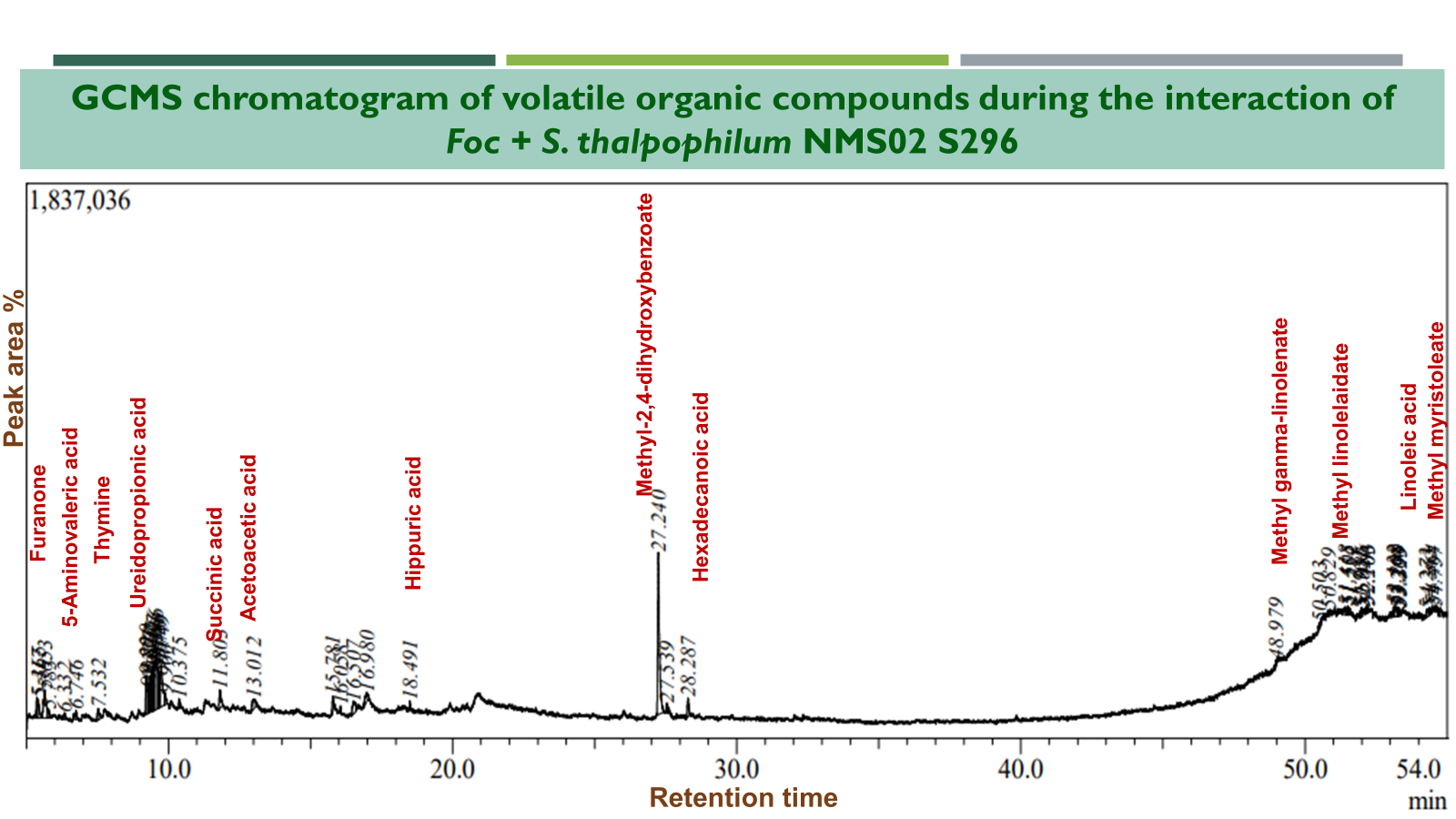


**Fig S5.** GC-MS chromatogram of VOCs/NVOCs produced by *Foc* + *S. thalpophilum* NMS02 S296


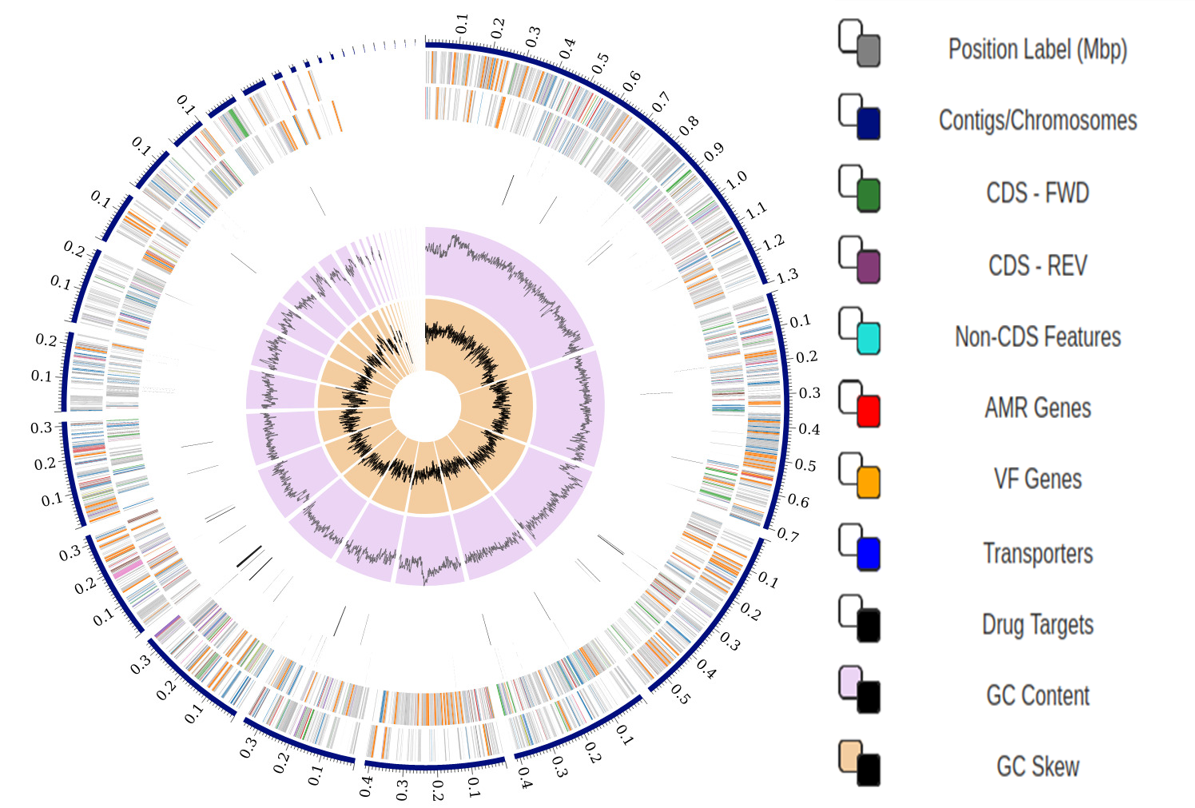


**Fig S6**. A circular graphical representation showcasing the distribution of genome annotations. The colors of the protein-coding sequences (CDS) on both the forward and reverse strands indicate the specific subsystem to which these genes belong. The circular representation includes various rings, starting from the outermost: contigs, CDS on the forward strand, CDS on the reverse strand, RNA genes, CDS with homology to known antimicrobial resistance genes, CDS with homology to known virulence factors, GC content, and GC skew.


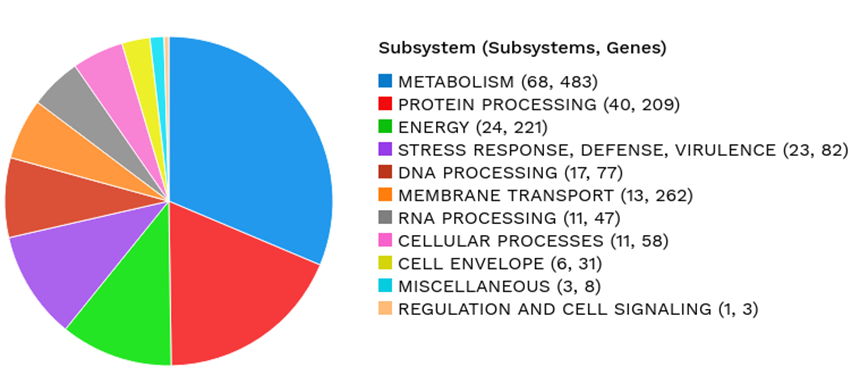


**Fig S7.** Subsystem superclass distribution of *S. thalpophilum* NMS02 S296


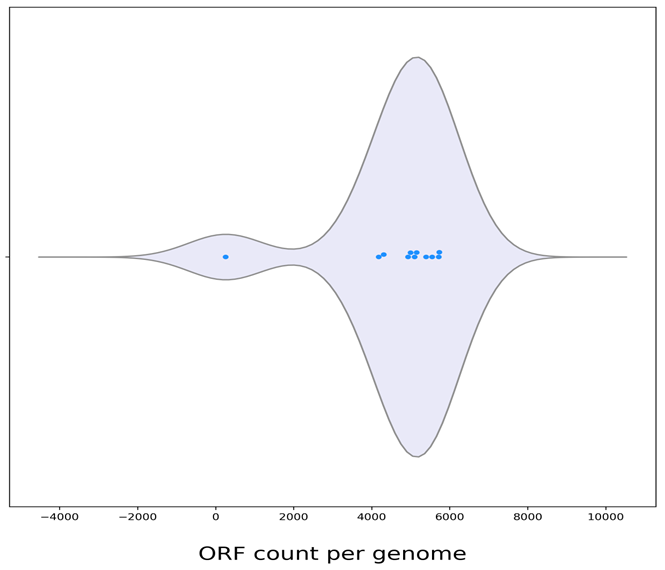


**Fig S8.** Violin plot representing the dispersion of the identified ORFs count per genome of *Sphingobacterium* spp.


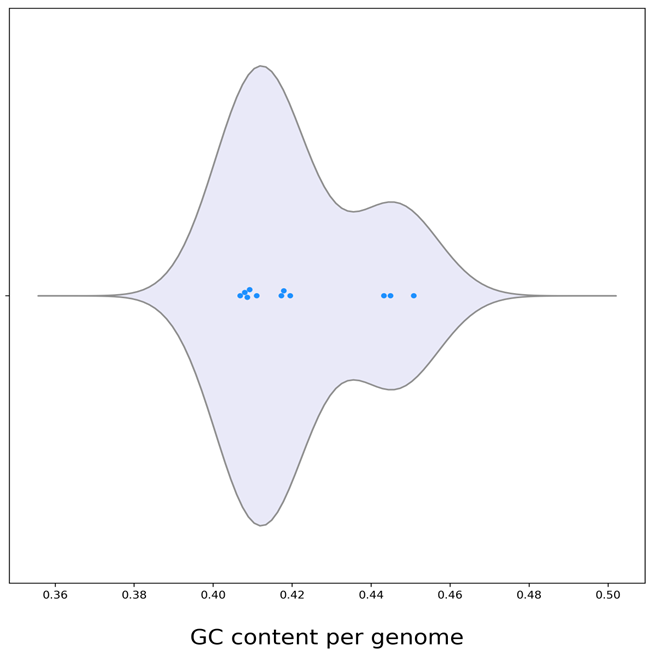


**Fig S9.** Violin plot representing the dispersion of the GC among genomes of *Sphingobacterium* spp.


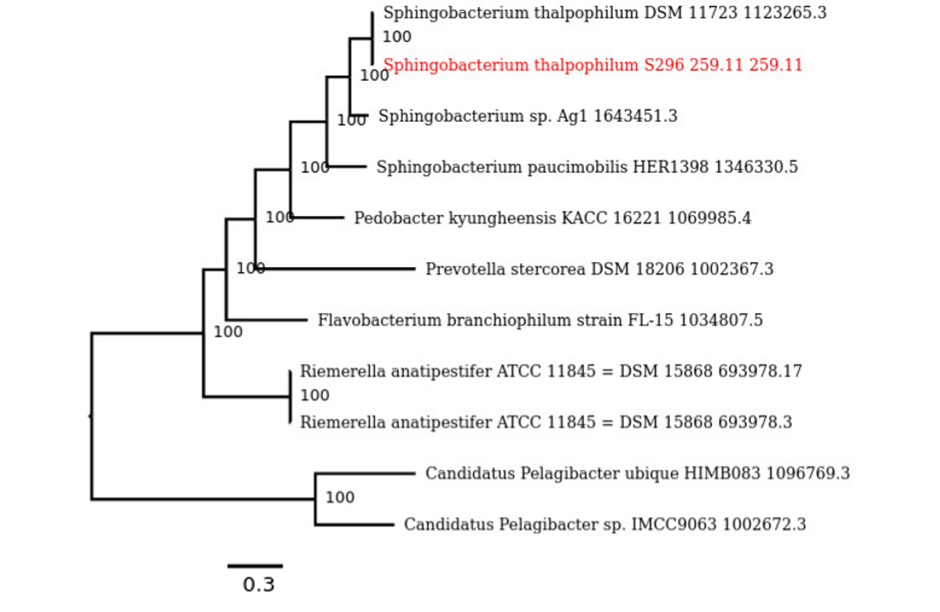


**Fig S10.** Phylogenetic analysis of the genome *S. thalpophilum* NMS02 S296**.**

**Fig S11.** Region-1 coding for secondary metabolite, betalactone in the genome of NMS02 S296

#
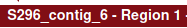


Location: 201,829 - 253,063 nt.**
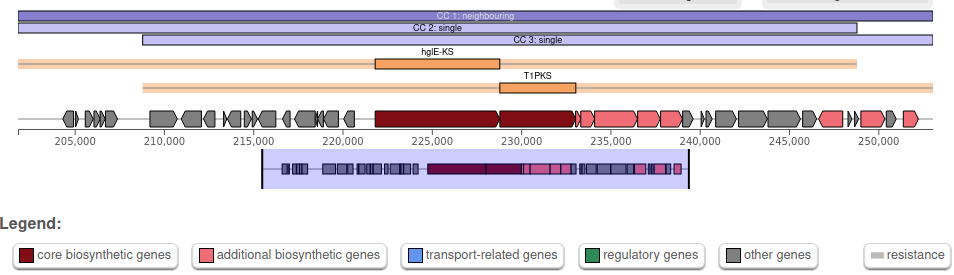
**


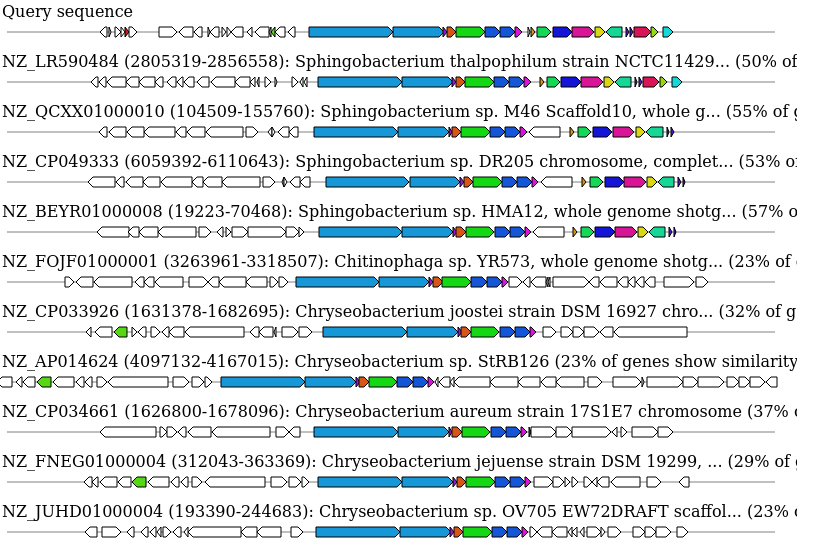


**Fig S12.** Region-2 coding for secondary metabolite, hglE-KS, T1PKS like in genome of NMS02 S296

#
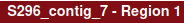


# Location: 312,749 - 324,911 nt

#
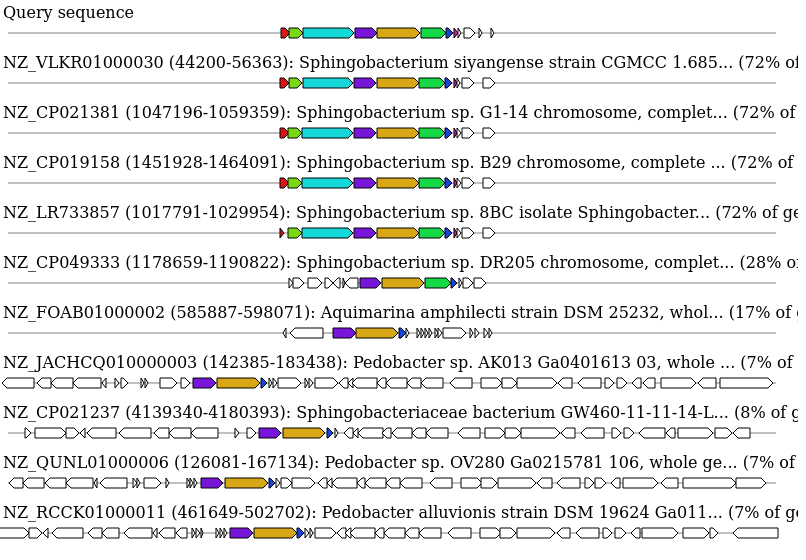

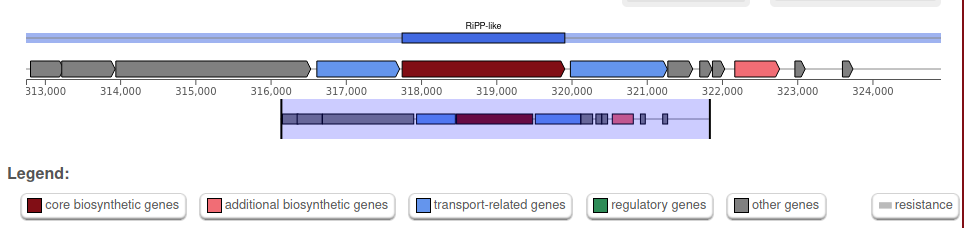


**Fig S13.** Region-3 coding for secondary metabolite, RiPP- like in genome of NMS02 S296


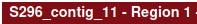


# Location: 1,840 - 22,676 nt


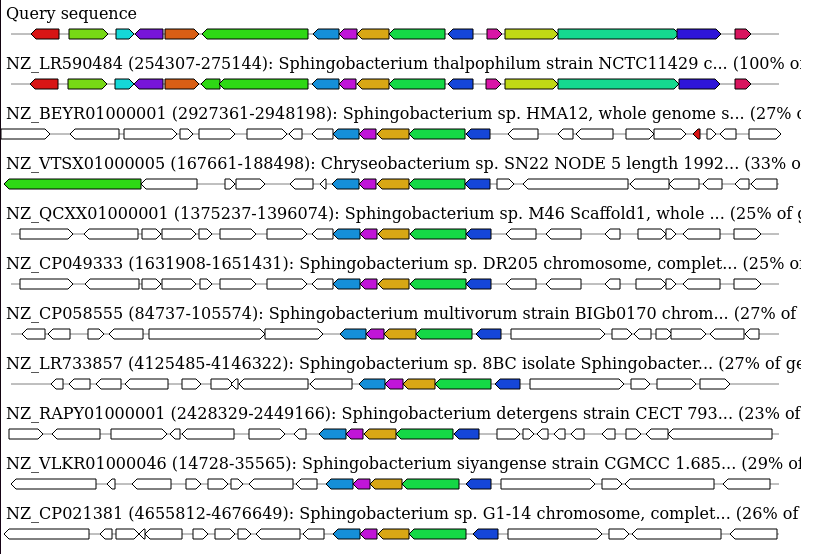

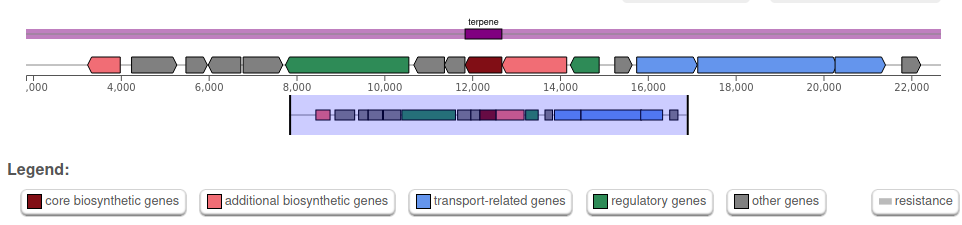


**Fig S14.** Region-4 coding for secondary metabolite, terpene in genome of NMS02 S296


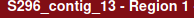


# Location: 120,442 - 138,522 nt


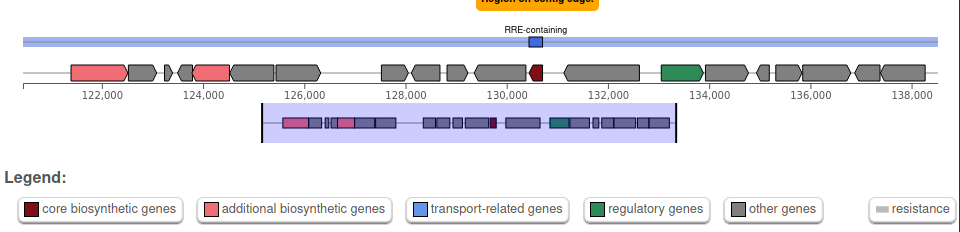


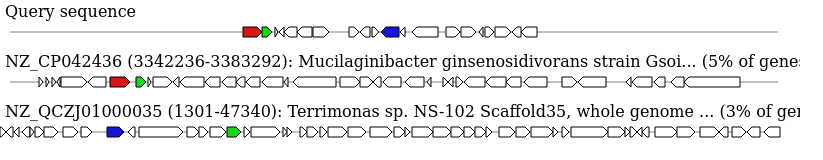


**Fig S15.** Region-5 coding for secondary metabolite, RRE containing in genome of NMS02 S296


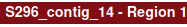


#
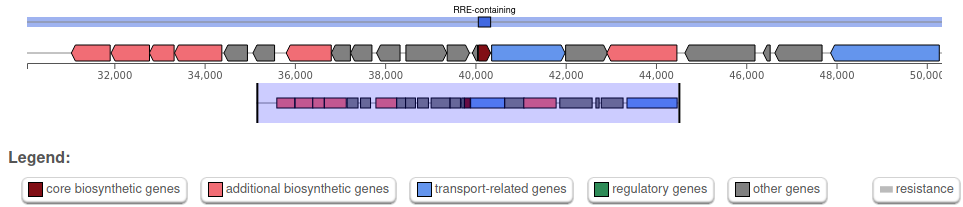
Location: 30,065 - 50,337 nt


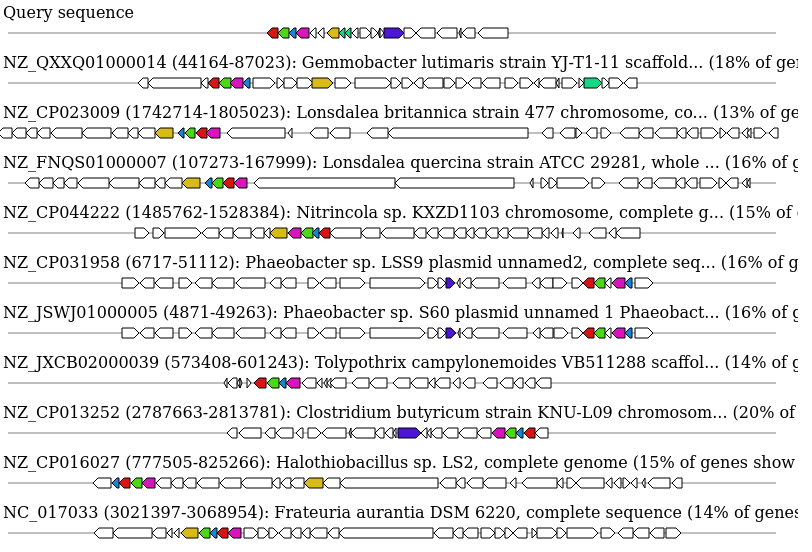


**Fig S16.** Region-6 coding for secondary metabolite, RRE containing in genome of NMS02 S296


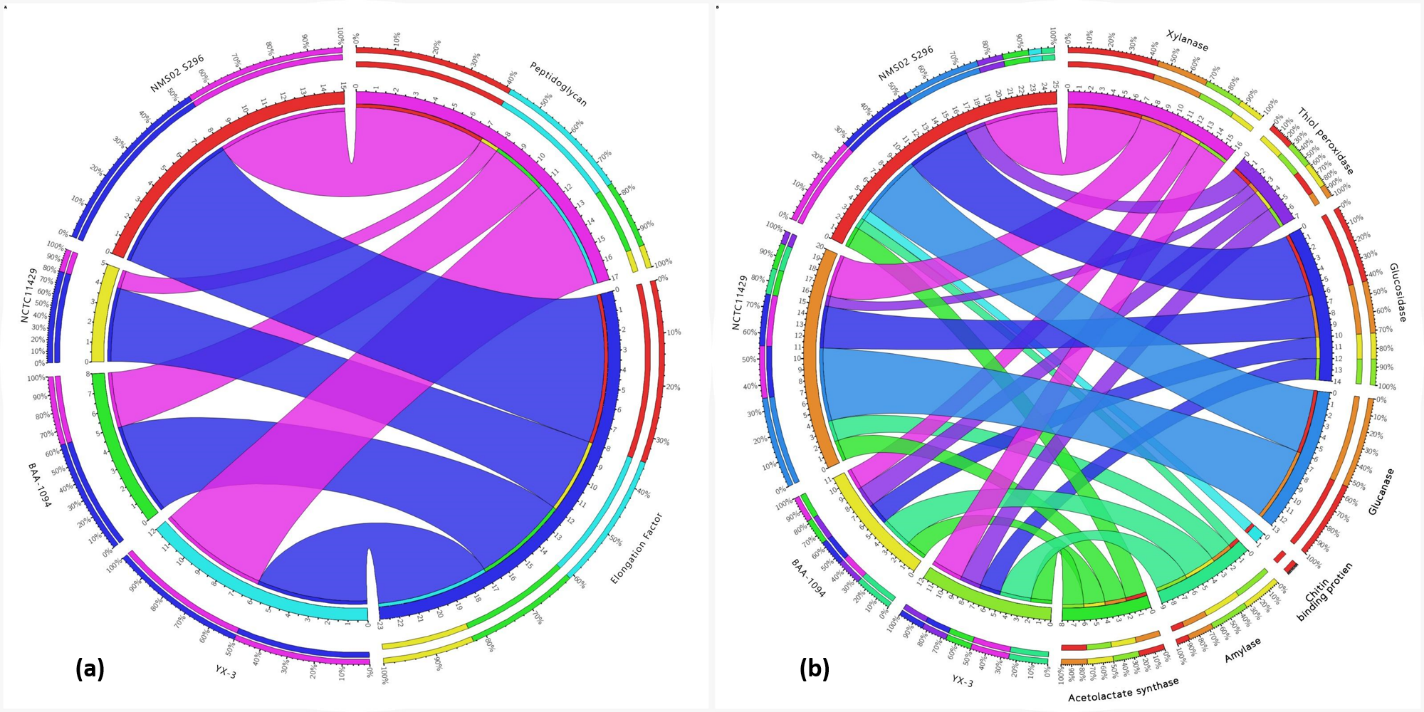


**Fig S17.** Circos plot depicting the various MAMP genes (Peptidoglycan and elongation factor) **(a)** and hydrolytic genes (amylase, xylanase, thiol peroxidase, glucosidase, glucanase, chitinase, chitin binding protein)  **(b)** in different strains of *S. thalpophilum*


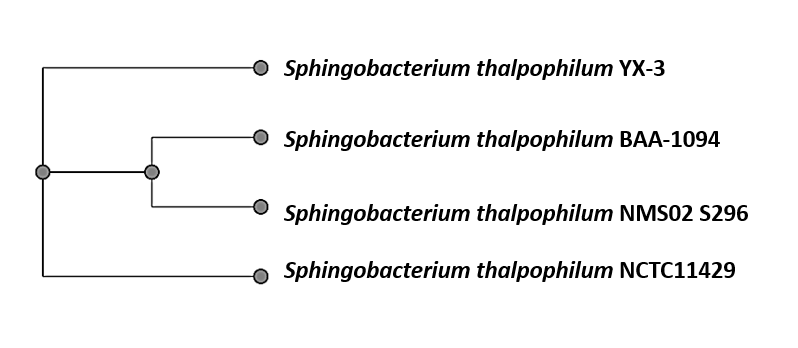


**Fig S18.** Phylogenetic relationship among four strains of *Sphingobacterium thalpophilum* constructed using the pan-based neighbour-joining method
